# The *Borrelia burgdorferi* RelA/SpoT Homolog and Stringent Response Regulate Survival in the Tick Vector and Global Gene Expression during Starvation

**DOI:** 10.1371/journal.ppat.1005160

**Published:** 2015-09-15

**Authors:** Dan Drecktrah, Meghan Lybecker, Niko Popitsch, Philipp Rescheneder, Laura S. Hall, D. Scott Samuels

**Affiliations:** 1 Division of Biological Sciences, University of Montana, Missoula, Montana, United States of America; 2 Department of Biology, University of Colorado, Colorado Springs, Colorado, United States of America; 3 Department of Biochemistry and Cell Biology, Max F. Perutz Laboratories, University of Vienna, Vienna, Austria; 4 Oxford NIHR Biomedical Research Centre, Wellcome Trust Centre for Human Genetics, University of Oxford, Oxford, United Kingdom; 5 Center for Integrative Bioinformatics Vienna, Max F. Perutz Laboratories, University of Vienna & Medical University of Vienna, Vienna, Austria; 6 Center for Biomolecular Structure and Dynamics, University of Montana, Missoula, Montana, United States of America; Medical College of Wisconsin, UNITED STATES

## Abstract

As the Lyme disease bacterium *Borrelia burgdorferi* traverses its enzootic cycle, alternating between a tick vector and a vertebrate host, the spirochete must adapt and persist in the tick midgut under prolonged nutrient stress between blood meals. In this study, we examined the role of the stringent response in tick persistence and in regulation of gene expression during nutrient limitation. Nutritionally starving *B*. *burgdorferi in vitro* increased the levels of guanosine tetraphosphate (ppGpp) and guanosine pentaphosphate (pppGpp), collectively referred to as (p)ppGpp, products of the bifunctional synthetase/hydrolase Rel_Bbu_ (RelA/SpoT homolog). Conversely, returning *B*. *burgdorferi* to a nutrient-rich medium decreased (p)ppGpp levels. *B*. *burgdorferi* survival in ticks between the larval and nymph blood meals, and during starvation *in vitro*, was dependent on Rel_Bbu_. Furthermore, normal morphological conversion from a flat-wave shape to a condensed round body (RB) form during starvation was dependent on Rel_Bbu_; *rel*_Bbu_ mutants more frequently formed RBs, but their membranes were compromised. By differential RNA sequencing analyses, we found that Rel_Bbu_ regulates an extensive transcriptome, both dependent and independent of nutrient stress. The Rel_Bbu_ regulon includes the *glp* operon, which is important for glycerol utilization and persistence in the tick, virulence factors and the late phage operon of the 32-kb circular plasmid (cp32) family. In summary, our data suggest that Rel_Bbu_ globally modulates transcription in response to nutrient stress by increasing (p)ppGpp levels to facilitate *B*. *burgdorferi* persistence in the tick.

## Author Summary

*Borrelia burgdorferi*, the spirochete responsible for causing Lyme disease, is maintained in nature via cycling between an *Ixodes* tick vector and a vertebrate host. The spirochete must adapt to and survive extreme nutrient deprivation, which may last months between blood meals, to persist in the midgut of the tick vector. How *B*. *burgdorferi* survives extended periods under such nutrient limitations has not been previously examined. In this study, we demonstrated that the stringent response, governed by Rel_Bbu_, which synthesizes and hydrolyzes the alarmones guanosine tetraphosphate and guanosine pentaphosphate (collectively termed (p)ppGpp), is necessary for persistence in the tick. Rel_Bbu_ was also required for survival during *in vitro* starvation and *rel*_Bbu_ mutants more readily formed round bodies, a morphological change recently implicated in persistence in the tick. These adaptations to nutrient limitations appear to be mediated by global changes in gene expression modulated by Rel_Bbu_ activity. Our results highlight an important role for Rel_Bbu_, and presumably (p)ppGpp, *in vivo* for persistence of a pathogen in its arthropod vector.

## Introduction

The Lyme disease spirochete *Borrelia burgdorferi* is maintained in an enzootic cycle involving ticks and vertebrates [1–3]. Since *B*. *burgdorferi* is not transovarially transmitted, the bacterium must be acquired by *Ixodes* larval ticks feeding on an infected mammal, the host reservoir. The larvae then molt into nymphs and the following year take another blood meal where spirochete transmission to naïve hosts may occur, completing the cycle. In order to navigate these transitions, *B*. *burgdorferi* must not only evade the host immune system, but also adapt to stressful environmental conditions in the arthropod by altering its gene expression [3,4]. A vital environmental factor in the tick midgut is available nutrients, including a carbon source, fatty acids, and nucleotides [5]. *B*. *burgdorferi* has limited biosynthetic capabilities and must scavenge nutrients from its environment [6,7]. As *B*. *burgdorferi* enters the larval midgut, along with the nutrient-rich blood meal, replication commences, dramatically increasing the number of spirochetes as the blood meal is consumed [8–11]. The midgut becomes depleted of nutrients within weeks [12]. *B*. *burgdorferi* may have to persist in this austere environment for months as the larvae molt into nymphs that will not feed until spring of the following year [12,13]. When the nymphs feed, the midgut milieu suddenly becomes rich in nutrients as the blood meal enters, triggering dormant *B*. *burgdorferi* to prepare to transmit to a new host [3,7,14,15]. Several *B*. *burgdorferi* gene products important for persistence in the tick have been identified [13,16], including BptA, a lipoprotein [17]; Dps/NapA/BicA, a bacterioferritin homolog [18]; GlpD (glycerol-3-phosphate dehydrogenase), an enzyme involved in glycerol metabolism [19]; and proteins involved in cyclic-dimeric-GMP (c-di-GMP) metabolism: Rrp1, a response regulator and diguanylate cyclase [20,21], Hk1, its cognate histidine kinase [22], PdeB, a phosphodiesterase [23], and PlzA, a c-di-GMP-binding protein [24]. However, none of these tick persistence factors have been shown to be associated with the requisite adaptation to nutrient limitations.

Bacteria adapt to nutritional limitations by adjusting their growth and modifying their physiology through the stringent response [25–29]. Global cell reprogramming induced during the stringent response is mediated by increases in the levels of two related nucleotide alarmones: guanosine pentaphosphate (pppGpp) and guanosine tetraphosphate (ppGpp), collectively abbreviated (p)ppGpp. These alarmones either directly or indirectly modulate transcription (rRNA, tRNA and stress regulons), translation, DNA replication, cell morphology, and numerous aspects of cellular physiology and metabolism [25,26,29–32]. In *Escherichia coli* and many other bacteria, (p)ppGpp levels are controlled by the enzymes RelA and SpoT, where RelA is a monofunctional synthetase and SpoT is a bifunctional synthetase/hydrolase. Some gram-positive bacteria contain both functional domains in a single enzyme termed Rel or RSH (RelA/SpoT homolog) [33]. Typically, RelA synthesizes ppGpp in response to amino acid starvation [34], while SpoT activity favors accumulation of (p)ppGpp in response to limiting fatty acids (FA) [35], phosphate [36], carbon [37], or iron [38]. Synthetase activity transfers a pyrophosphate (PP_i_) from ATP to either GDP to form ppGpp (and AMP) or to GTP to form pppGpp (and AMP). SpoT and SpoT-like domains hydrolyze either pppGpp to GTP and PP_i_ or ppGpp to GDP and PP_i_. Until recently, pppGpp and ppGpp have been considered essentially equivalent regarding the cellular response elicited; however, studies in *E*. *coli* have shown that subtleties of the stringent response depend not only on the overall alarmone concentration, but also the relative amounts of pppGpp and ppGpp [39–41].

The effects of (p)ppGpp on transcription are complex and global [42–44]. (p)ppGpp affects the activity of RNA polymerase (RNAP) both directly and indirectly through DksA (DnaK suppressor) [45–47]; these interactions can increase or decrease transcription depending on the specific sequence near the promoter. Typically genes involved in vegetative cell growth whose expression is mediated by σ^70^ (RpoD) are downregulated while those involved in the stress response and/or adaptations to nutrient limitations are upregulated. Indirectly, increasing (p)ppGpp levels affects sigma factor selectivity as more RNAP is released from some σ^70^-promoters allowing alternative sigma factors, such as σ^S^ (RpoS), to bind RNAP, further shifting the program of gene expression [25,29,45]. In addition, pppGpp production consumes GTP and thereby decreases the cellular GTP concentration, which is significant enough in some bacteria, such as *B*. *subtilis*, to inhibit transcription initiation [48].

The influence of (p)ppGpp levels on growth and survival during nutrient stress is also intimately entwined with various aspects of virulence in numerous pathogens [49,50]. The alarmone transduces signals from environmental cues to indicate when conditions are favorable to replicate, transmit, or persist. For example, (p)ppGpp regulates expression of the alternative sigma factor FliA in *Legionella pneumophila* to control replication and transmission in host cells [51], and modulates the activity of transcription factors HilA and SlyA in *Salmonella enterica* serovar Typhimurium to induce expression of *Salmonella* pathogenicity islands 1 and 2 [52,53]. Therefore, while (p)ppGpp induces general physiological and metabolic changes to adapt to nutrient stress, the alarmone also triggers intracellular processes specific for microbial virulence in response to different environments [25,28,29,49].

Adaptive morphological changes in response to environmental stresses are also regulated by (p)ppGpp in many bacteria [31]. Elevated (p)ppGpp levels induced by starvation correlated with *Mycobacterium smegmatis* converting from bacilli to coccoid forms [54]. *Myxococcus xanthus* requires (p)ppGpp in order to initiate the pathway leading to myxospore formation during nutrient-limiting conditions [55]. Abolishing (p)ppGpp production in *Helicobacter pylori* causes the premature formation of coccoid forms [56]. Notably, *B*. *burgdorferi* undergoes conversion to a condensed non-motile morphology termed a round body (RB) during starvation *in vitro* and, to a certain extent, in the midgut of the flat tick, although the role of (p)ppGpp in this process has not been previously evaluated [57–60].

The *B*. *burgdorferi* gene product BB0198 (Rel_Bbu_) contains domains homologous to RelA and SpoT; Rel_Bbu_ has been shown to be responsible for (p)ppGpp production and *rel*_Bbu_ can heterologously complement an *E*. *coli relA/spoT* double mutant [61–63].The conditions that modulate (p)ppGpp levels and the role of this important intracellular messenger in adaptation of *B*. *burgdorferi* to nutrient stress remain scarcely studied. In this work, we examine the *in vivo* role of *rel*_Bbu_ in the tick-mouse model of Lyme disease as well as the *in vitro* role in survival and regulation of global gene expression by comparative RNA sequencing (RNA-seq).

## Results

### Levels of (p)ppGpp increase in *B*. *burgdorferi* during nutrient starvation

**Fig 1:**
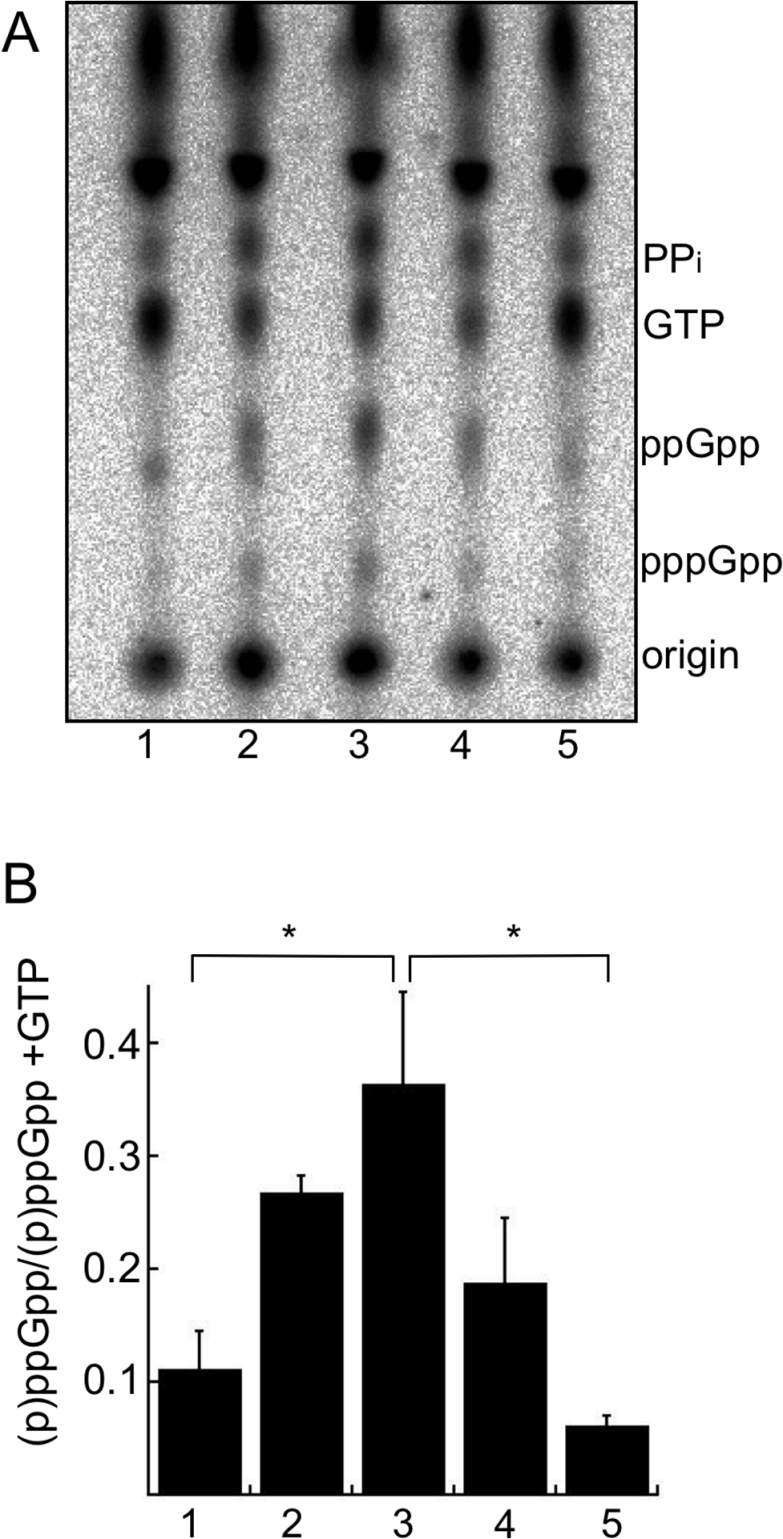
Starvation of *B*. *burgdorferi* in RPMI increases (p)ppGpp levels. (A) Analysis of radiolabeled nucleotides in wild-type strain B31-5A4 grown in ^32^P-orthophosphate. Cultures were grown to late log phase in BSK + RS (lane 1) and then starved in RPMI culture medium for 30 min (lane 2) or 6 h (lane 3) and then shifted back to BSK + RS for 10 min (lane 4) or 2 h (lane 5). At each time point, nucleotides were extracted and separated by TLC. TLC plates were dried, exposed to a phosphor screen and visualized using a phosphorimager. (B) Quantification of (p)ppGpp levels by densitometry. Values represent the mean of three independent experiments, performed as described in A (lanes 1–5), expressed as (p)ppGpp normalized to (p)ppGpp + GTP. Error bars represent SEM. Asterisks indicate *P* < 0.05 as determined by one-way ANOVA and a Tukey’s *post-hoc* test.

To adapt to the stress of nutrient starvation, bacteria increase (p)ppGpp levels, which invokes substantial physiological changes to aid survival. We assayed if *B*. *burgdorferi* increases (p)ppGpp levels during starvation by shifting *in vitro* cultures from the normal growth medium, Barbour-Stoenner-Kelly II medium containing 6% rabbit serum (BSK + RS), to a starvation medium (RPMI containing no serum). This definition of starvation was used because shifting cells to RPMI removes many of the nutrients present in BSK and was previously used to mimic nutrient stress and starvation conditions in the tick [60,63,64]. The starvation medium notably lacks rabbit serum, as well as neopeptone, yeastolate, *N*-acetylglucosamine, and bovine serum albumin. *B*. *burgdorferi* strain B31-5A4 (wild type) was grown in BSK + RS and labeled with ^32^P-orthophosphate (20 μCi ml^-1^). Cultures were starved in RPMI or starved and then recovered in BSK + RS, aliquots were collected at each time point, nucleotides were extracted and samples were resolved by thin layer chromatography (TLC). Both pppGpp and ppGpp levels increased during starvation (Fig 1A, lanes 1–3), with a significant increase in (p)ppGpp observed after 6 h in RPMI compared to cells growing in BSK + RS (Fig 1B, 1 and 3). Concomitant with increased (p)ppGpp levels was a decrease in cellular GTP levels and an increase in pyrophosphate (PP_i_) levels (Fig 1A, lanes 2 and 3). Spots corresponding to GTP and PP_i_ in the cell extracts were determined by running α-^32^P -GTP and ^32^PP_i_ as standards (S1 Fig). To determine if (p)ppGpp levels changed during *B*. *burgdorferi* recovery from starvation, we starved ^32^P-labeled cells for 6 h in RPMI before returning them to BSK + RS for 10 min (Fig 1A, lane 4) or 2 h (Fig 1A, lane 5). (p)ppGpp levels decreased significantly in *B*. *burgdorferi* returned to nutrient-rich medium (BSK + RS) for 2 h (Fig 1B, 5) compared to 6 h in RPMI (Fig 1B, 3). PP_i_ levels also decreased and GTP levels increased during recovery from starvation (Fig 1A, lanes 4 and 5). Therefore, *B*. *burgdorferi* modulates (p)ppGpp levels in response to nutrient stress, although the specific extracellular signal(s) remain to be identified for this pathogen.

**Fig 2:**
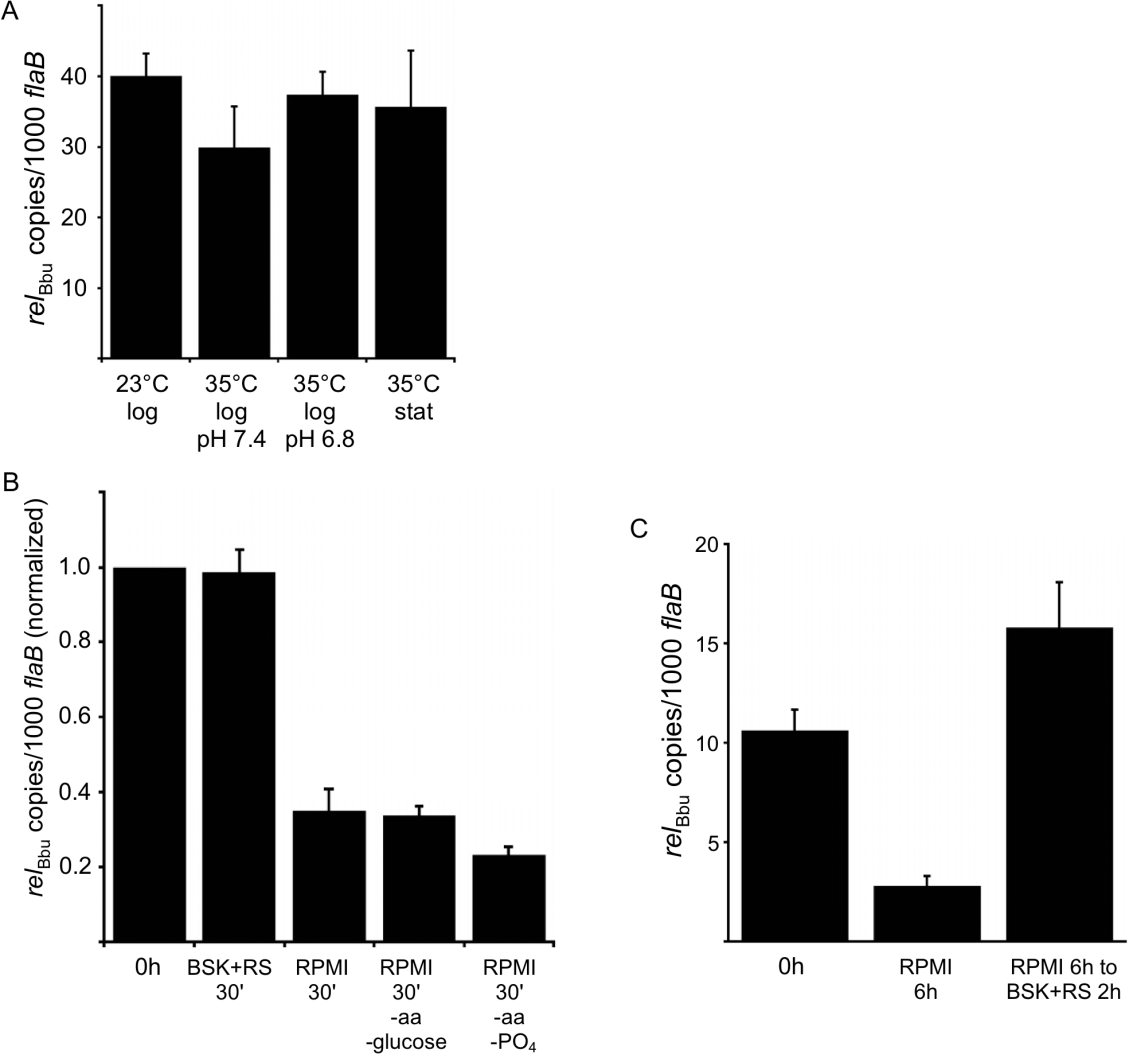
*rel*_Bbu_ transcript levels change in response to nutrient levels. (A) Temperature, growth phase and pH do not affect *rel*_Bbu_ transcript levels. *B*. *burgdorferi* strain B31-5A4 was grown in BSK + RS at 23°C or 35°C to log or stationary (stat) phase at pH 7.4 or 6.8 before RNA was isolated, cDNA synthesized and *rel*_Bbu_ transcript levels measured by qRT-PCR. (B) Starvation conditions reduce *rel*_Bbu_ transcript levels. *B*. *burgdorferi* strain B31-5A4 was grown in BSK + RS at 35°C to late log phase before cultures were collected and shifted to RPMI, RPMI without amino acids (aa) and glucose, RPMI without amino acids (aa) and phosphate (PO_4_), or returned to BSK + RS at 35°C. After 30 min, RNA was isolated, cDNA synthesized and *rel*_Bbu_ transcript levels measured by qRT-PCR. Values are normalized to *rel*_Bbu_ transcript levels at the beginning of each experiment (0 h). (C) *B*. *burgdorferi* strain B31-5A4 was grown in BSK + RS at 35°C (0 h), starved in RPMI for 6 h or starved in RPMI for 6 h, and then shifted back to BSK + RS for 2 h. At each time point, *rel*_Bbu_ transcript levels were measured as described above. Each value is the mean of three independent experiments and error bars represent the SEM.

Since Rel_Bbu_ is predicted to be a bifunctional enzyme responsible for both (p)ppGpp synthesis and hydrolysis, the expected response of *rel*_Bbu_ gene expression to environmental signals and growth conditions was not obvious. Temperature, increasing from 23°C to 35°C, and pH, lowering from 7.4 to 6.8, are both signals that have been proposed to regulate gene expression during transmission of *B*. *burgdorferi* during tick feeding [65–67]. We assayed *rel*_Bbu_ gene expression in response to these environmental conditions by qRT-PCR and found no significant difference in *rel*_Bbu_ transcript levels, suggesting that temperature, pH and growth phase do not control expression of the *rel*_Bbu_ gene *in vitro* (Fig 2A). We next examined if nutrient levels affect the amount of *rel*_Bbu_ transcript by starving *B*. *burgdorferi* in RPMI medium and comparing *rel*_Bbu_ transcript levels to those in normal growth medium (BSK + RS) by qRT-PCR. *rel*_Bbu_ transcript levels decreased about threefold when compared to *flaB* transcript levels, which actually increased slightly, within 30 min of starvation compared to either the original culture or cells collected by centrifugation and returned to BSK + RS (Fig 2B). There was no significant difference in the observed threefold decrease in *rel*_Bbu_ transcript levels in cells incubated in RPMI and those starved in RPMI lacking amino acids, glucose or phosphate, indicating that these three components are not *in vitro* environmental cues that regulate *rel*_Bbu_ transcript levels (Fig 2B). When *B*. *burgdorferi* was starved in RPMI for longer times (6 h), *rel*_Bbu_ transcript levels remained depressed, but recovered to levels similar to those observed before starvation after returning cultures to complete medium (BSK + RS) (Fig 2C). The reduction in *rel*_Bbu_ transcript was unexpected since (p)ppGpp levels increased during starvation, but may reflect a strategy to decrease the potential to hydrolyze (p)ppGpp by decreasing the amount of bifunctional Rel_Bbu_. The relationship between *rel*_Bbu_ transcript levels, Rel_Bbu_ protein levels, and coordination of synthetase and hydrolase activity requires further investigation.

**Fig 3:**
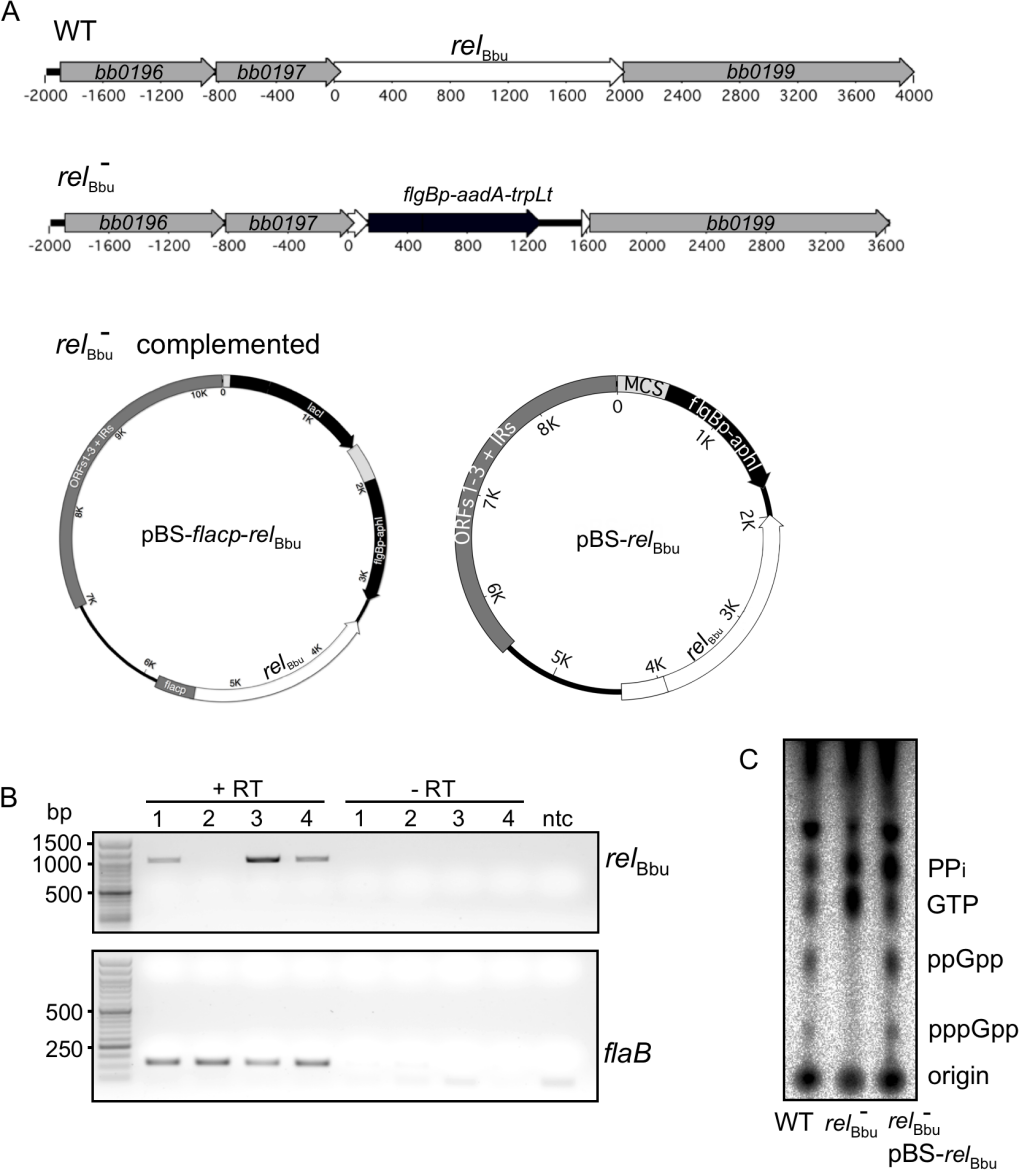
Mutation and complementation of *rel*_Bbu_ in *B*. *burgdorferi*. (A) The *rel*_Bbu_ mutant (*rel*_Bbu_^-^) was constructed by replacing the *rel*_Bbu_ gene in strain B31-5A4 with the streptomycin resistance gene *aadA* fused to the *B*. *burgdorferi flgB* promoter and the *B*. *subtilis trpL* terminator. The *rel*_Bbu_ mutant was complemented in *trans* by transformation with the *Borrelia* shuttle vector pBSV2 containing the *rel*_Bbu_ gene fused to either the *flacp* inducible promoter (pBS-*flacp*-*rel*_Bbu_) or its native promoter (pBS-*rel*_Bbu_). (B) RT-PCR analysis of RNA isolated from wild-type (lane 1), *rel*_Bbu_^-^ (lane 2), *rel*_Bbu_^-^ pBS-*flacp*-*rel*_Bbu_ (lane 3), or *rel*_Bbu_^-^ pBS-*rel*_Bbu_ (lane 4) strains. Samples were incubated with (+RT) or without (-RT) reverse transcriptase, and *rel*_Bbu_ and *flaB* transcripts were detected by PCR using primer pairs rsh 981F/rsh 1984R and flaB 423F/flaB 542R, respectively. Products were separated on 1% (*rel*_Bbu_) or 2% (*flaB*) agarose gels and stained with ethidium bromide. ntc = no template control. (C) Production of (p)ppGpp in the wild-type (WT), *rel*_Bbu_^*-*^ and *rel*_Bbu_^*-*^ pBS-*rel*_Bbu_ (*rel*_Bbu_^-^ comp) strains. ^32^P-labeled cultures were grown to log phase, shifted to RPMI for 6 h and nucleotides were extracted and analyzed by TLC.

To assay if the *rel*_Bbu_ gene product was responsible for the increase in (p)ppGpp levels observed during starvation, we disrupted the *rel*_Bbu_ gene with a streptomycin/spectinomycin resistance cassette [68] to generate a *rel*_Bbu_ mutant strain (Fig 3A). The *rel*_Bbu_ mutant strain was complemented using two different strategies: the *rel*_Bbu_ gene was either fused to the *flac* promoter [69] and inserted into the shuttle vector pBSV2 [70] to yield pBS-*flacp-rel*_Bbu_ or cloned along with 365 upstream nucleotides, which contain the native promoter [61], and inserted into pBSV2 to yield pBS-*rel*_Bbu_ (Fig 3A). The *rel*_Bbu_ transcript was present in the wild-type and complemented strains, but absent in the mutant strain by RT-PCR (Fig 3B). To examine if (p)ppGpp production was dependent on the *rel*_Bbu_ gene product, wild-type, *rel*_Bbu_ mutant and complemented (*rel*_Bbu_^-^ pBS-*rel*_Bbu_) strains were labeled with ^32^P-orthophosphate, shifted to starvation medium for 6 h and nucleotides analyzed as described for Fig 1. The wild-type and complemented strains produced pppGpp and ppGpp under starvation conditions while the *rel*_Bbu_ mutant strain did not (Fig 3C).

### Survival during nutrient starvation depends on *rel*_Bbu_

**Fig 4:**
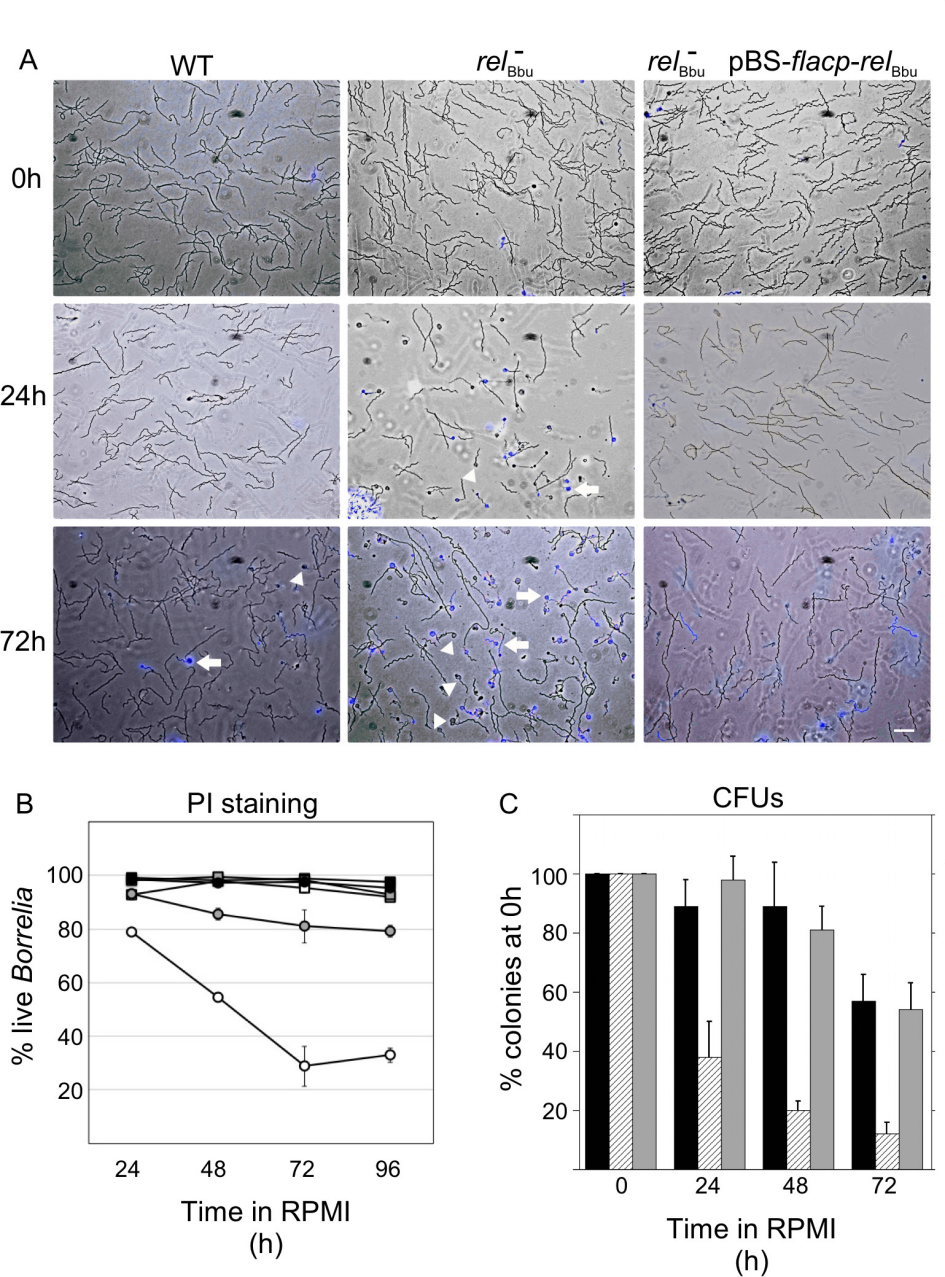
Survival of *B*. *burgdorferi* during starvation conditions *in vitro* depends on *rel*_Bbu_. Strains were grown to late log phase in BSK + RS (0 h) before shifting to RPMI for 24 h or 72 h. Wild-type (WT), *rel*_Bbu_ mutant (*rel*_Bbu_^*-*^), and *rel*_Bbu_^*-*^ pBS-*flacp*-*rel*_Bbu_ cells were stained with PI and visualized by DIC and fluorescence microscopy. Images are overlays of DIC and fluorescence images with PI shown in blue. Arrowheads are RBs that did not stain with PI and arrows point to PI-stained RBs. Scale bar = 10 μm. (B) The percentage of live spirochetes as quantified by PI staining following incubation in RPMI for various times (24 h to 96 h). Black squares represent WT in BSK + RS, black circles represent WT in RPMI, white squares represent *rel*_Bbu_^-^ in BSK + RS, white circles represent *rel*_Bbu_^-^ in RPMI, gray squares represent *rel*_Bbu_^*-*^ pBS-*flacp*-*rel*_Bbu_ in BSK + RS, and gray circles represent *rel*_Bbu_^*-*^ pBS-*flacp*-*rel*_Bbu_ in RPMI. Values are the means of three independent experiments with at least 100 spirochetes counted for each time point and error bars represent SEM. (C) Wild-type (black bars), *rel*_Bbu_^-^ (hatched bars) and *rel*_Bbu_^-^ pBS-*rel*_Bbu_ (gray bars) strains were grown in BSK + RS to late log phase before shifting to RPMI. To quantify the number of live *B*. *burgdorferi* at the time points indicated, samples of each culture were plated in semi-solid BSK, allowed to grow for two weeks and colonies enumerated to yield colony forming units (CFUs). Each value represents the mean of three independent experiments normalized to the initial number of colonies before shifting to RPMI (0 h). Error bars represent SEM.

To examine if *B*. *burgdorferi* survival during nutrient starvation is *rel*_Bbu_-dependent, we assayed cell viability *in vitro* [71,72]. Wild-type, *rel*_Bbu_ mutant and complemented (*rel*_Bbu_^-^ pBS-*flacp*-*rel*_Bbu_) strains were grown to late log phase in BSK + RS medium before shifting to RPMI medium for 0, 24 or 72 h. At these times, cultures were incubated with propidium iodide (PI), which stains cells with compromised membranes (i.e., dead cells). Live cultures were wet-mounted and both differential interference contrast (DIC) and fluorescence images were collected and overlaid. As a positive control to ensure that PI stained nonviable *B*. *burgdorferi*, cells were heat-killed by incubating at 94°C for 5 min before PI staining: we found that 99% of cells were stained with PI following heat treatment. The *rel*_Bbu_ mutant strain did not survive as well as the wild-type and complemented strains when incubated in RPMI for 24 and 72 h, as seen by the increased number of PI-stained spirochetes (blue) (Fig 4A). Notably, many *rel*_Bbu_ mutant cells assumed a condensed spherical morphology, termed round bodies (RB) [57,58], during starvation. RBs were more frequently seen in the *rel*_Bbu_ mutant than in wild-type or complemented strains. Many of the RBs stained with PI (Fig 4A, arrows), but others did not (Fig 4A, arrowheads), suggesting that some RBs remained viable. By quantifying the number of PI-stained cells, we found that survival of the *rel*_Bbu_ mutant was significantly decreased compared to wild-type and complemented strains throughout starvation (Fig 4B). Similar results were obtained when viability was quantified by enumerating the colony forming units of strains plated in semi-solid BSK following a time course of starvation. Again, the survival of the *rel*_Bbu_ mutant (Fig 4C; hatched bars) during starvation was compromised compared to the wild-type and complemented strains (Fig 4C; black bars and gray bars, respectively). Therefore, *rel*_Bbu_ has a crucial function for survival of *B*. *burgdorferi* under nutrient stress.

### Formation of round bodies (RBs) is regulated by *rel*_Bbu_

**Fig 5:**
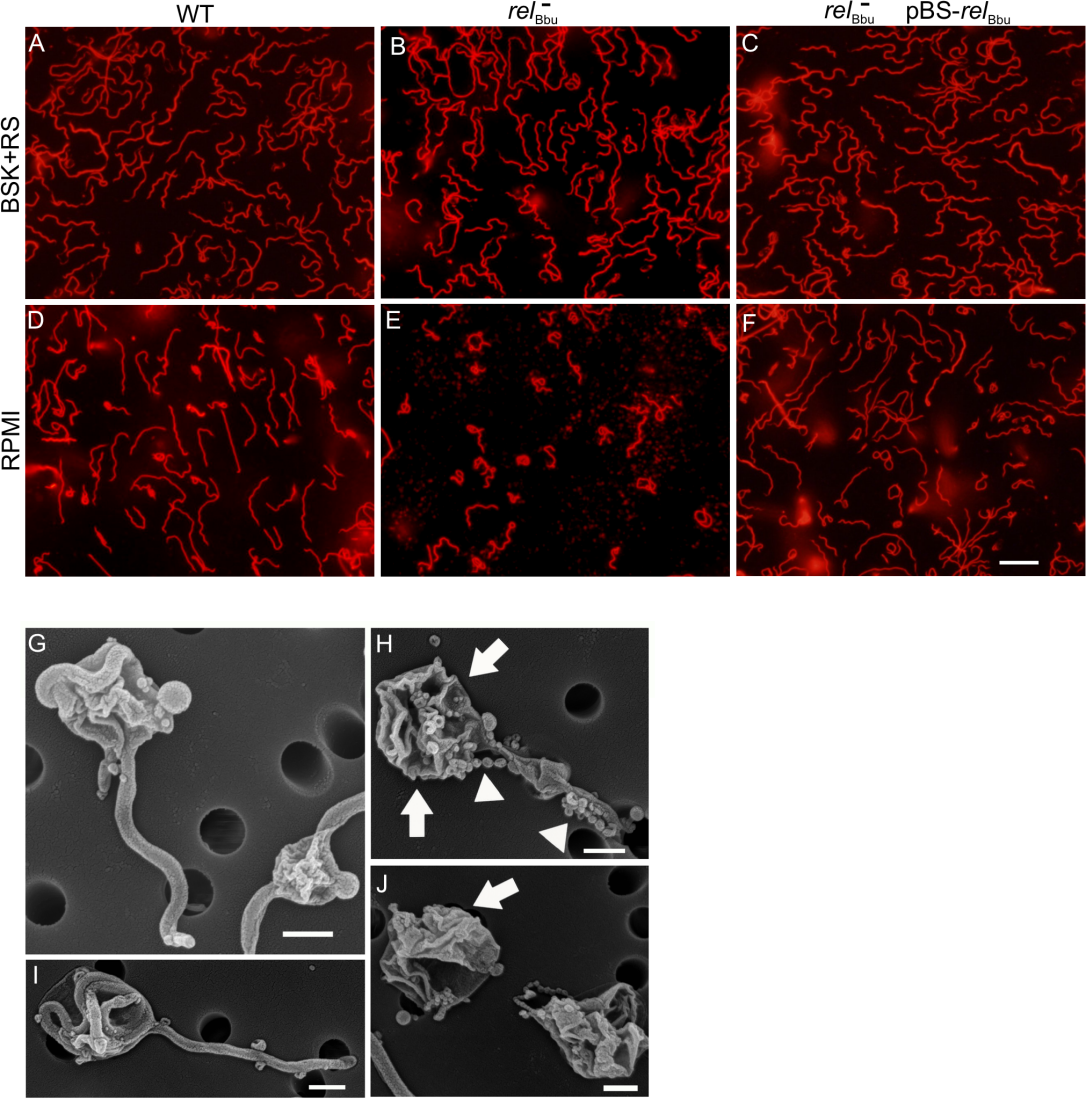
*rel*_Bbu_ regulates round body formation under starvation conditions *in vitro*. Fluorescence microscopy of live *B*. *burgdorferi* wild-type (WT), *rel*_Bbu_ mutant (*rel*_Bbu_^*-*^), and *rel*_Bbu_^*-*^ pBS-*rel*_Bbu_ strains grown in BSK + RS to late log phase (A-C) or shifted to RPMI for 2 days (D-F) and stained with WGA-Alexa Fluor 594. Scale bar = 10 μm. Scanning electron microscopy of *rel*_Bbu_^*-*^ strains after shifting to RPMI for 2 days (G-J). Arrows indicate folded and disrupted membranes and arrowheads indicate membrane beading. Scale bar = 600 nm.

To further investigate the role of *rel*_Bbu_ in RB formation induced by starvation, we developed a method to simply and rapidly visualize live *Borrelia* cultures without using fixative (such as acetone or paraformaldehyde), which can affect morphology, and without expressing fluorescent proteins. Incubating *B*. *burgdorferi* with wheat germ agglutinin attached to a fluorophore (WGA-Alexa Fluor 594) rapidly labels essentially all of the bacteria (S2 Fig) by binding to sialic acid and *N*-acetylglucosamine residues on the surface, so they are readily visible by fluorescence microscopy. Wild-type, *rel*_Bbu_ mutant and complemented strains were grown in BSK + RS or shifted to RPMI for 48 h and then stained with WGA-Alexa Fluor 594. Cells were wet-mounted on slides and immediately imaged. While all three strains had the same flat-wave morphology characteristic of the spirochete when grown in BSK + RS (Fig 5A–5C), the majority of *rel*_Bbu_ mutant cells converted to the RB phenotype under starvation conditions (Fig 5E) compared to wild-type and complemented cells (Fig 5D and 5F). Wild-type and complemented strains still formed RBs, but at a lower frequency compared to the *rel*_Bbu_ mutant. To more closely examine the morphology of the *rel*_Bbu_ mutant RBs, samples that were starved for 48 h were subjected to scanning electron microscopy. There appeared to be two types of RBs formed from the *rel*_Bbu_ mutant strain: one in which the membrane appeared intact and smooth as the cylinder condensed and contracted into a ball (Fig 5G and 5I), and another in which the membrane was disrupted and folded (Fig 5H and 5J, arrows) and showed membrane blebbing (Fig 5H, arrowheads).

**Fig 6:**
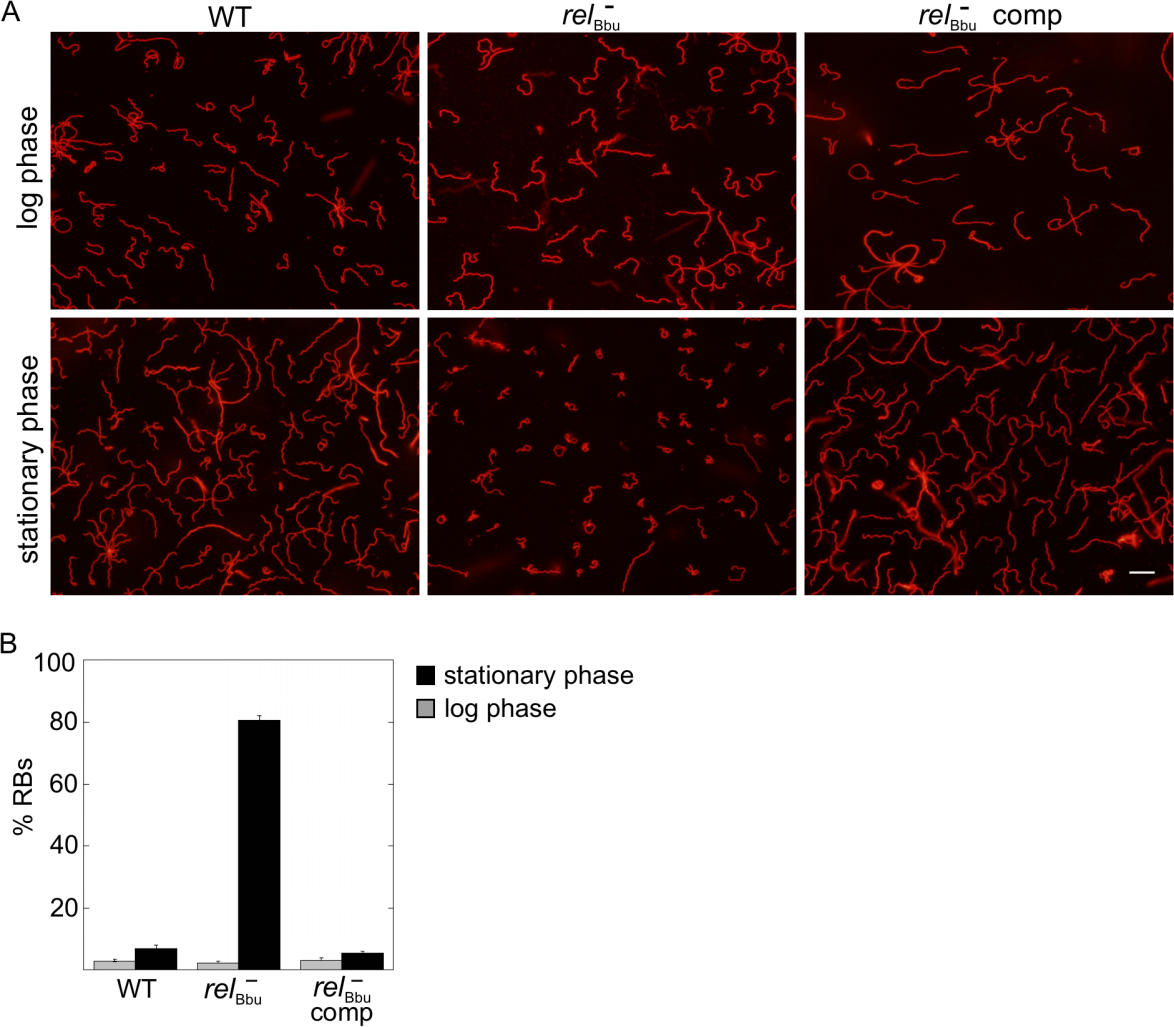
*rel*_Bbu_ affects *B*. *burgdorferi* morphology in stationary phase. (A) Live cell microscopy of wild-type (WT), *rel*_Bbu_ mutant (*rel*_Bbu_^-^) and *rel*_Bbu_^-^ pBS-*flacp*-*rel*_Bbu_ (*rel*_Bbu_^-^ comp) strains grown to late log phase or late stationary phase in BSK + RS before staining with WGA-Alexa Fluor 594. Scale bar = 10 μm. (B) RB quantification in log (gray bars) and stationary (black bars) phase. Each value represents the mean of three independent experiments; error bars represent SEM.

The morphology of *B*. *burgdorferi* cells grown *in vitro* was also examined as cells transitioned to stationary phase. The *rel*_Bbu_ mutant strain again condensed to form RBs more often than the wild-type and complemented strains when cells were grown in BSK + RS well into stationary phase (~3 x 10^8^ cells ml^-1^) as visualized by fluorescence microscopy of WGA-Alexa Fluor 594-stained cells (Fig 6). These results are similar to those found during starvation of the *rel*_Bbu_ mutant strain and suggest the stationary phase environment may induce a similar response in *B*. *burgdorferi*. Taken together, these data suggest that Rel_Bbu_ controls the decision to undergo, and possibly the program of, RB formation.

### Rel_Bbu_ is not required for murine infection by needle inoculation

Enzymes that metabolize (p)ppGpp have been shown in other bacteria to regulate numerous virulence factors, some of which mediate host interactions including, but not limited to, immune evasion, motility, transmission, and replication [49]. To test if *rel*_Bbu_ in *B*. *burgdorferi* is required for mammalian infection, mice were intradermally needle-inoculated with either 10^5^ or 10^6^ of wild-type, *rel*_Bbu_ mutant or complemented cells. Tissues were collected three and five weeks post injection, cultured for *B*. *burgdorferi* and monitored by dark-field microscopy for the presence of spirochetes. The *rel*_Bbu_ mutant strain was able to infect mice and disseminate to the ear, ankle joints and bladder (Table 1), indicating that *rel*_Bbu_ is not required for mammalian infection in the murine model of Lyme disease.

**Table 1:** Mouse infectivity of the *B*. *burgdorferi rel*_Bbu_ mutant.

Route	Strain	Ear^b^	Ankle^b^	Bladder^b^
Needle^a^	wild type	3/3	3/3	3/3
	*rel*_*Bbu*_^-^	3/3	3/3	3/3
	*rel*_*Bbu*_^-^ pBS-*rel*_*Bbu*_	3/3	3/3	3/3
Nymph^c^	wild type	7/7	7/7	7/7
	*rel*_*Bbu*_^-^	3/12	3/12	1/12
	*rel*_*Bbu*_^-^ pBS-*rel*_*Bbu*_	3/3	3/3	3/3

^a^ Intradermal injection with 10^5^ cells.

^b^ Tissues collected five weeks post-injection or post nymph feeding and cultured in BSK medium. Figures are the number of infected mice/number of mice tested.

^c^ Five nymphs infected with the indicated strains were allowed to feed to repletion on naïve mice.

The natural route of mammalian infection is transmission by tick bite. To examine if Rel_Bbu_ is required for tick transmission, naïve larvae were fed to repletion on mice infected with wild-type, *rel*_Bbu_ mutant or complemented strains. After molting to nymphs, five ticks were placed on a mouse and allowed to feed to repletion, and mice were assessed for infection three and five weeks later as described above. Wild-type and complemented strains were transmitted from nymphs to all infested mice, while only 3 of 12 mice were infected by nymphs carrying the *rel*_Bbu_ mutant strain (three independent experiments with one out of four mice infected in each experiment; Table 1). There are two explanations, which are not mutually exclusive: Rel_Bbu_ plays a role in tick transmission, but is not absolutely required and/or transmission is compromised due to low levels of *rel*_Bbu_ mutants in nymphs due to a persistence defect.

### Rel_Bbu_ is required for persistence in the tick vector

**Fig 7:**
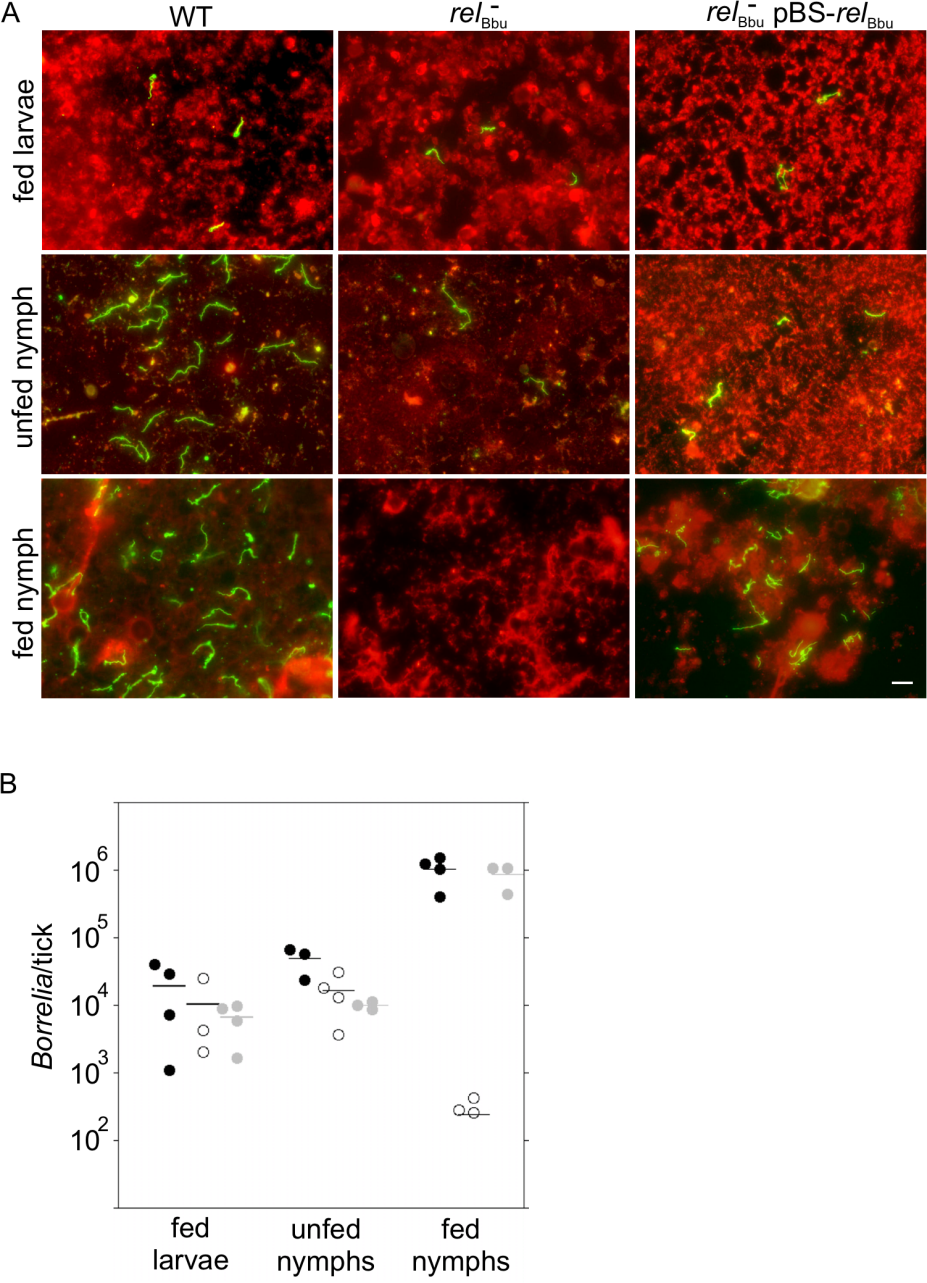
Tick persistence depends on *rel*_Bbu_. (A) Immunofluorescence microscopy of ticks allowed to feed on mice infected with wild-type (WT), *rel*_Bbu_ mutant (*rel*_Bbu_^-^) or *rel*_Bbu_^-^ pBS-*rel*_Bbu_ strains. Ticks were dissected and fixed on slides one week after naïve larvae had fed to repletion (fed larvae) or after larvae had molted to nymphs (unfed nymph) or one week after nymphs had fed to repletion on uninfected mice (fed nymph). Samples were processed for IF microscopy using rabbit polyclonal anti-*B*. *burgdorferi* antibodies followed by goat anti-rabbit Alexa Fluor 488 antibodies to visualize spirochetes (green). Tick cells were visualized by staining with WGA-Alexa Fluor 594 (red). Scale bar = 10 μm. (B) Quantification of *Borrelia* in ticks that had fed on mice infected with wild-type (black circles), *rel*_Bbu_^-^ (white circles) or *rel*_Bbu_^-^ pBS-*rel*_Bbu_ (gray circles) strains. Total DNA was isolated from larvae that had fed to repletion (fed larvae) or after larvae had molted to nymphs (unfed nymph) or one week after nymphs had fed to repletion on uninfected mice (fed nymph). The number of *B*. *burgdorferi* genome equivalents per tick was determined by qPCR using TaqMan primers/probe to *flaB*. The difference between the number of WT and *rel*_Bbu_^-^ in fed nymphs was statistically significant (*P* = 0.018) by a one-way ANOVA with a Tukey’s *post hoc* test.

Our *in vitro* data suggest that *rel*_Bbu_ is important for survival during starvation. We hypothesized that *rel*_Bbu_ is required for persistence in the tick vector between blood meals, where *B*. *burgdorferi* experiences nutrient stress [12,13]. To test this hypothesis, naïve *Ixodes scapularis* larvae were allowed to feed to repletion on mice infected by needle inoculation with wild-type, *rel*_Bbu_ mutant or complemented strains as described above. *B*. *burgdorferi* persistence in the tick was assayed by immunofluorescence (IF) microscopy (Fig 7A). At each stage (fed larvae, flat nymphs and fed nymphs), six ticks were dissected on a slide, fixed and processed for IF microscopy using anti-*B*. *burgdorferi* antibodies followed by Alexa Fluor 488 secondary antibodies (green); tick cells were labeled with WGA-Alexa Fluor 594 (red). Fed larvae acquired all strains to a similar degree (Fig 7A, top row). However, the *rel*_Bbu_ mutant, while still present in flat nymphs, did not persist after the nymphs fed on uninfected mice (Fig 7A, middle column).

To confirm these results by another method, the *Borrelia* load per tick was quantified at each stage by qPCR. Total DNA was isolated and qPCR was performed using primers/probe to the *B*. *burgdorferi flaB* gene. The number of spirochetes per tick in fed larvae and flat nymphs was not significantly different in wild type- (black circles), *rel*_Bbu_ mutant—(white circles) or *rel*_Bbu_^*-*^pBS-*rel*_Bbu_- (gray circles) infected ticks (Fig 7B). Again, the *rel*_Bbu_ mutant did not persist from flat to fed nymphs: there were significantly fewer spirochetes detected in nymphs infected with the *rel*_Bbu_ mutant strain compared to nymphs infected with the wild-type strain. Persistence was restored in the complemented strain (Fig 7B).

### Transcriptome changes during starvation and recovery from starvation

To examine global transcriptional changes occurring during nutrient stress, the transcriptomes of wild-type *B*. *burgdorferi* grown to stationary phase, starved for 6 h, and recovered from starvation were compared by RNA-seq. Two independent experiments were performed, comparisons were combined, and both DEseq and EdgeR analyses were used to calculate the significance of differential gene expression (see Materials and Methods). Only genes whose transcript levels were significantly changed (*P* < 0.05) and varied by twofold or greater were considered to be affected by nutrient stress or dependent on Rel_Bbu_ (S1–S10 Tables). Furthermore, only sequences that mapped uniquely to the genome were included and the differential expression of each significantly regulated gene was manually inspected and pseudogenes removed from the lists. These analyses likely underestimate the number of affected genes, particularly of the cp32s, due to the extreme sequence similarity in some regions of the genome [6,73,74].

**Fig 8:**
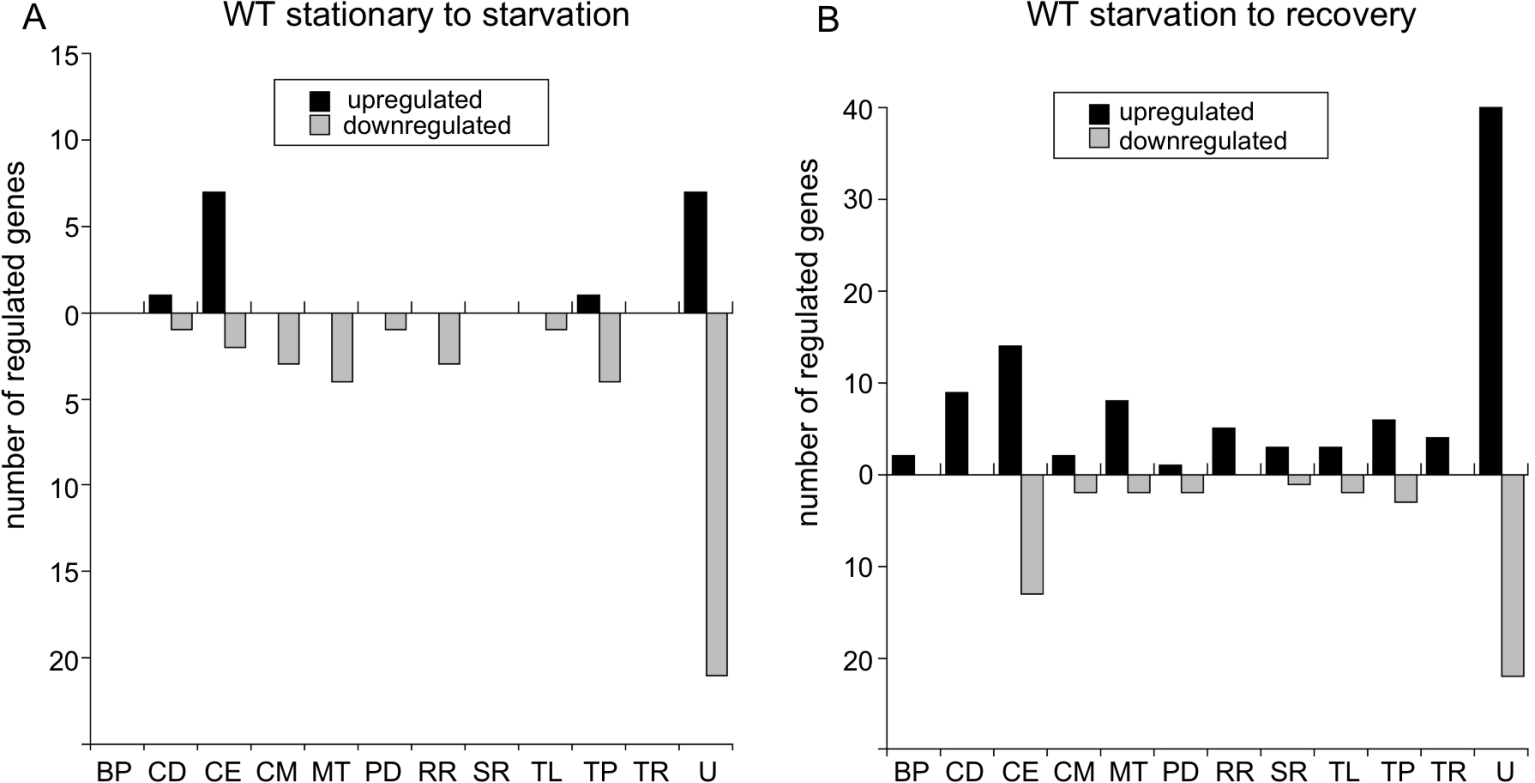
Changes in transcript levels in wild-type *B*. *burgdorferi* during nutrient stress. Genes significantly differentially expressed more than twofold as determined by RNA-seq of wild-type B31-5A4 (A) shifted from stationary phase to starvation (6 h RPMI) and (B) in recovery (2 h BSK + RS) from starvation. The number of genes upregulated (black bars) and downregulated (gray bars) are divided by functional category using the following abbreviations: BP, bacteriophage; CD, cell division; CE, cell envelope; CM, chemotaxis and motility; MT, metabolism; PD, protein degradation; RR, DNA replication and repair; SR, stress response; TL, translation; TP, transporter proteins; TR, transcription and transcriptional regulation; and U, unknown.

When wild-type cultures were starved (6 h in RPMI), only 16 genes were upregulated compared to cells in stationary phase, with the majority encoding cell envelope proteins and lipoproteins (CE) or encoding proteins, mostly hypothetical, of unknown function (U) (Fig 8A, black bars). Notably, *glpF* (*bb0240*), encoding the glycerol uptake facilitator, and *dbpB* (*bba25*), encoding a decorin-binding protein, were both significantly upregulated (S1 Table). Forty genes were downregulated during starvation of wild-type cells with the majority, again, encoding hypothetical proteins (Fig 8A, gray bars and S2 Table).

During recovery of the wild-type strain from starvation (6 h in RPMI medium followed by 2 h in BSK II + RS), more genes were upregulated (97 genes) than were downregulated (47 genes) (Fig 8B; S3 and S4 Tables). The majority of upregulated and downregulated genes during recovery encoded proteins of unknown function. The other functional categories containing numerous upregulated genes were: cell division (CD); cell envelope and lipoproteins, including the antigenic variation expression locus *vlsE* (*bbf0041*); and metabolism (MT), including the genes *pfs*, *metK* and *luxS* from the *bb0374*-*bb0377* operon [75] (Fig 8B and S3 Table). *luxS* was also downregulated during starvation of wild type (S2 Table), suggesting transcript levels of this gene respond positively and negatively to nutrient levels. *csrA* (*bb0184*), which encodes the carbon storage regulator, is induced under conditions mimicking mammalian infection [76] and was upregulated during recovery. Additionally, two genes encoding proteins in the master pathway regulating genes required for infectivity [4], a sensory transduction histidine kinase (*hk2*; *bb0764*) and the alternative sigma factor σ^54^ (*rpoN*; *bb0450*), were induced during recovery (S3 Table). Interestingly, the expression of the key transcriptional regulator of this pathway, *rpoS*, was not upregulated more than twofold.

During recovery of the wild-type strain from starvation, the majority of downregulated genes encoded products of unknown function or in the cell envelope and lipoproteins category (Fig 8B, gray bars), including the outer membrane protein P66 (*p66*; *bb0603*), which binds β chain integrins and has porin activity [16]. Two genes from the glycerol metabolism (*glp*) operon were downregulated: *glpF* and *glpK* (*bb0241*), encoding glycerol kinase (S4 Table). Thus, *glpF* was induced during starvation in wild-type cells and repressed during recovery from starvation, results consistent with a proposed role in the tick [19]. *dps*/*napA*/*bicA* (*bb0690*), another gene whose product is important for persistence in the tick [18], was also repressed during recovery (S4 Table). Therefore, in wild-type cells, some of the genes involved in infection tend to be upregulated during recovery from starvation while the genes that play a role for persistence in the tick tend to be repressed.

### *B*. *burgdorferi* genes upregulated by Rel_Bbu_

**Fig 9:**
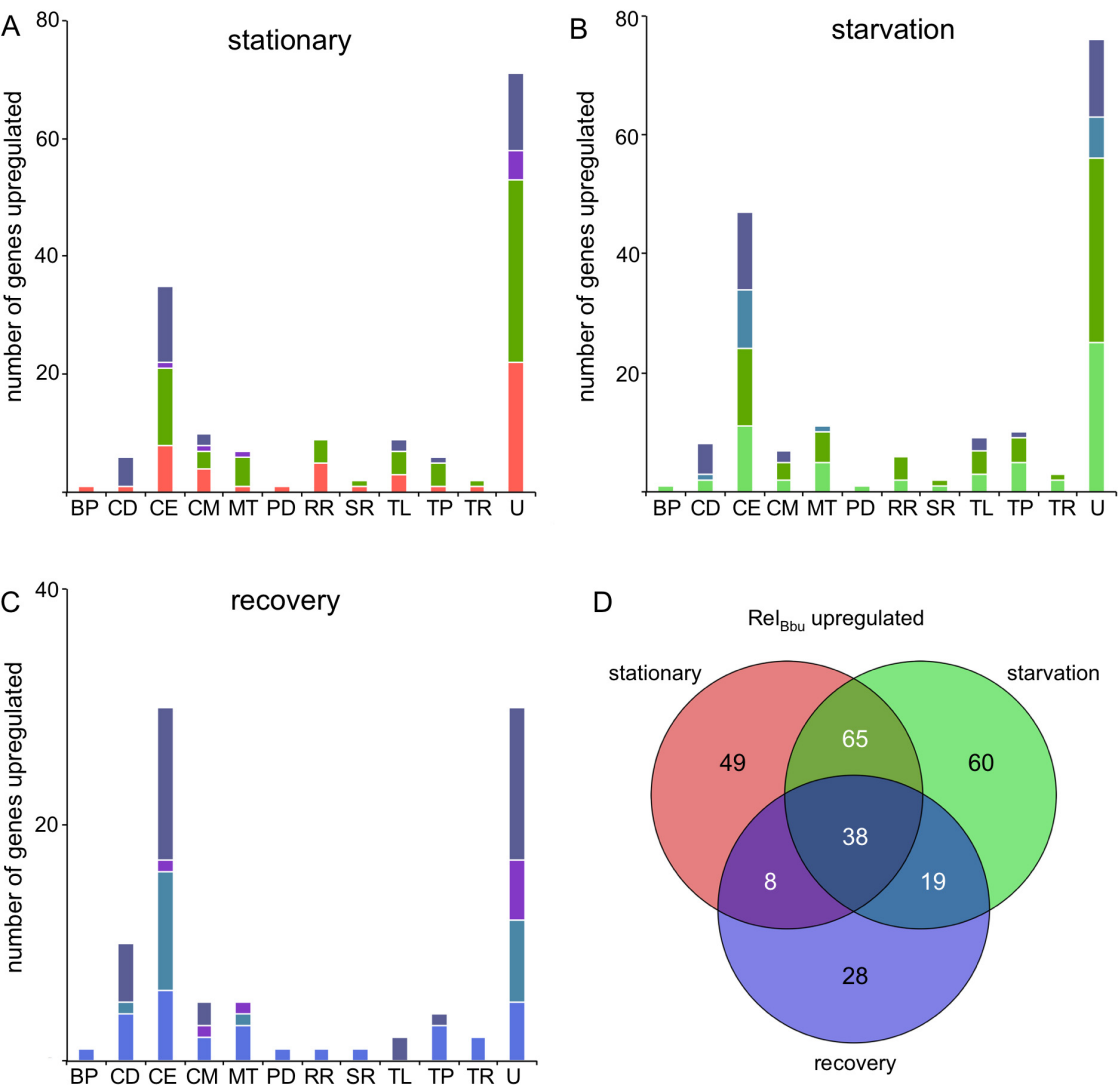
Rel_Bbu_-upregulated genes during nutrient stress. The number of genes significantly Rel_Bbu_-upregulated (higher in wild type than *rel*_Bbu_^-^) greater than twofold as determined by RNA-seq in (A) stationary phase, (B) starvation and (C) recovery from starvation. Bars are color-coded based on the color scheme assigned in (D) for unique and overlapping conditions that upregulate genes. (D) Venn diagram of the number of genes Rel_Bbu_-upregulated in stationary phase, starvation and recovery from starvation. Functional gene category abbreviations are the same as in Fig 8.

To examine the role Rel_Bbu_ and (p)ppGpp have in global gene regulation during nutrient stress, we compared the transcriptomes of wild-type and *rel*_Bbu_ mutant strains by RNA-seq in stationary phase, during starvation, and in recovery from starvation from two independent experiments (twofold cutoff; *P* < 0.05) as described above. Rel_Bbu_ directly or indirectly at least doubled the transcript levels (i.e., higher expression in the wild-type transcriptome than in the *rel*_Bbu_ mutant transcriptome) of 160 genes at stationary phase, 182 genes during starvation, and 93 genes during recovery from starvation (Fig 9D and S5–S7 Tables). About a third of the genes upregulated under each condition were unique to that condition. Thirty-eight genes were upregulated in all three conditions, suggesting that Rel_Bbu_ is important for their expression independent of extracellular nutrients. Cells in stationary phase and under starvation conditions shared more upregulated genes than were shared between starvation and recovery or stationary phase and recovery (Fig 9D). Rel_Bbu_-dependent changes in transcript levels measured by RNA-seq were validated by qRT-PCR under stationary phase, starvation and recovery conditions (S3 Fig). Rel_Bbu_-mediated upregulation and repression were confirmed in the majority of genes and conditions, but qRT-PCR generally underestimated the differences between wild-type and *rel*_Bbu_ mutant strains found by RNA-seq.

To gain insight into the biological processes influenced by Rel_Bbu_ and (p)ppGpp in *B*. *burgdorferi*, we plotted the number of upregulated genes by functional category and shared response to the three conditions. For example, the 44 cell envelope and lipoprotein genes Rel_Bbu_ upregulated under starvation conditions are fairly evenly distributed as unique to starvation, shared between stationary and starvation, shared between starvation and recovery, and shared by all three conditions (Fig 9B). The majority of Rel_Bbu_-upregulated genes in all three conditions encode products of unknown function or cell envelope and lipoprotein genes (Fig 9A–9C). Closer examination of the Rel_Bbu_-dependent transcriptome reveals the biological processes controlled by Rel_Bbu_ and the stringent response. Many of the upregulated cell envelope and lipoprotein genes encode products that are known to bind host extracellular matrix proteins, including decorin (*bba24* and *bba25*), laminin (*bbq47*), fibronectin (*bbk32*, *bbm27*, *bbp27*, and *bb0347*), and collagen (*bba33*), suggesting that Rel_Bbu_ has a role in the interaction of *B*. *burgdorferi* with its host (S5–S7 Tables). In addition, the *vlsE* gene is upregulated by Rel_Bbu_ under all three conditions, indicating some regulation by Rel_Bbu_ that is independent of nutrient levels. *vlsE* is the expression site of a recombination system used for antigenic variation of the surface lipoprotein VlsE that allows *B*. *burgdorferi* to evade the host immune system during infection [77,78]. *dbpA* and *dbpB* were upregulated by Rel_Bbu_ during starvation (S6 Table), but not during stationary phase (S5 Table), raising the possibility that regulation of these genes responds more dramatically to (p)ppGpp than other genes upregulated by Rel_Bbu_. *dbpBA* transcript levels also remain elevated during the recovery phase (S7 Table), implying an intricate and subtle relationship between Rel_Bbu_, (p)ppGpp, and gene expression that modulates host-pathogen interactions.

Transcript levels of *ospC* (*bbb19*), encoding an outer membrane lipoprotein, were Rel_Bbu_-upregulated during starvation and recovery but not stationary phase. OspC is essential for mammalian infection and its transcription is regulated by a complex dual sigma factor cascade involving RpoN and RpoS [3,4,16]. While levels of *rpoN* were not increased by Rel_Bbu_, *rpoS* (*bb0771*) levels were upregulated in stationary phase, but unchanged during starvation and recovery. Additionally, the gene encoding the DNA-binding protein BosR (*bb0647*), which is an important regulator of RpoS-mediated virulence gene expression [4,79–81], was induced by Rel_Bbu_ during stationary phase and starvation (S5 and S6 Tables).

Glycerol metabolism genes in the *glp* operon were also regulated by Rel_Bbu_. *glpF* was upregulated in stationary phase and during starvation, while *glpK* was upregulated during starvation. Glycerol and the products of the *glp* operon have been shown to function in *B*. *burgdorferi* growth *in vitro* and tick persistence [19,20,82]. Our data support these observations and suggest a mechanism linking changing nutritional cues, gene regulation and control of carbon utilization.

A group of genes encoding an oligopeptide transporter system was also upregulated by Rel_Bbu_. *B*. *burgdorferi* lacks the ability to synthesize most amino acids and is thought to scavenge peptides from the environment to fulfill this need [6,7]. The genes encoding oligopeptide binding proteins were upregulated in stationary phase (*oppA1* and *oppA2*), starvation (*oppA1*, *oppA2* and *oppA3*), and recovery (*oppA2* and *oppA5*) (S5–S7 Tables). Our findings also agree with a previously reported role for Rel_Bbu_ in regulation of these transport proteins [83]. These data, along with the results from Iyer et al. [84] that expression of *oppA1* and *oppA3* was higher in ticks compared to mice, support a role for Rel_Bbu_ in the tick. In addition, *oppA5* was shown by Iyer et al. to be expressed at higher levels in mice than ticks [84], while we discovered that its expression was increased only during recovery from starvation (S7 Table). Expression of the genes encoding other components of the oligopeptide transport system such as permeases (*oppB1*/*oppC1* and *oppB2*/*oppC2*) and ATP-binding proteins (*oppD* and *oppF*) were not Rel_Bbu_-dependent.

### *B*. *burgdorferi* genes repressed by a Rel_Bbu_-mediated mechanism

**Fig 10:**
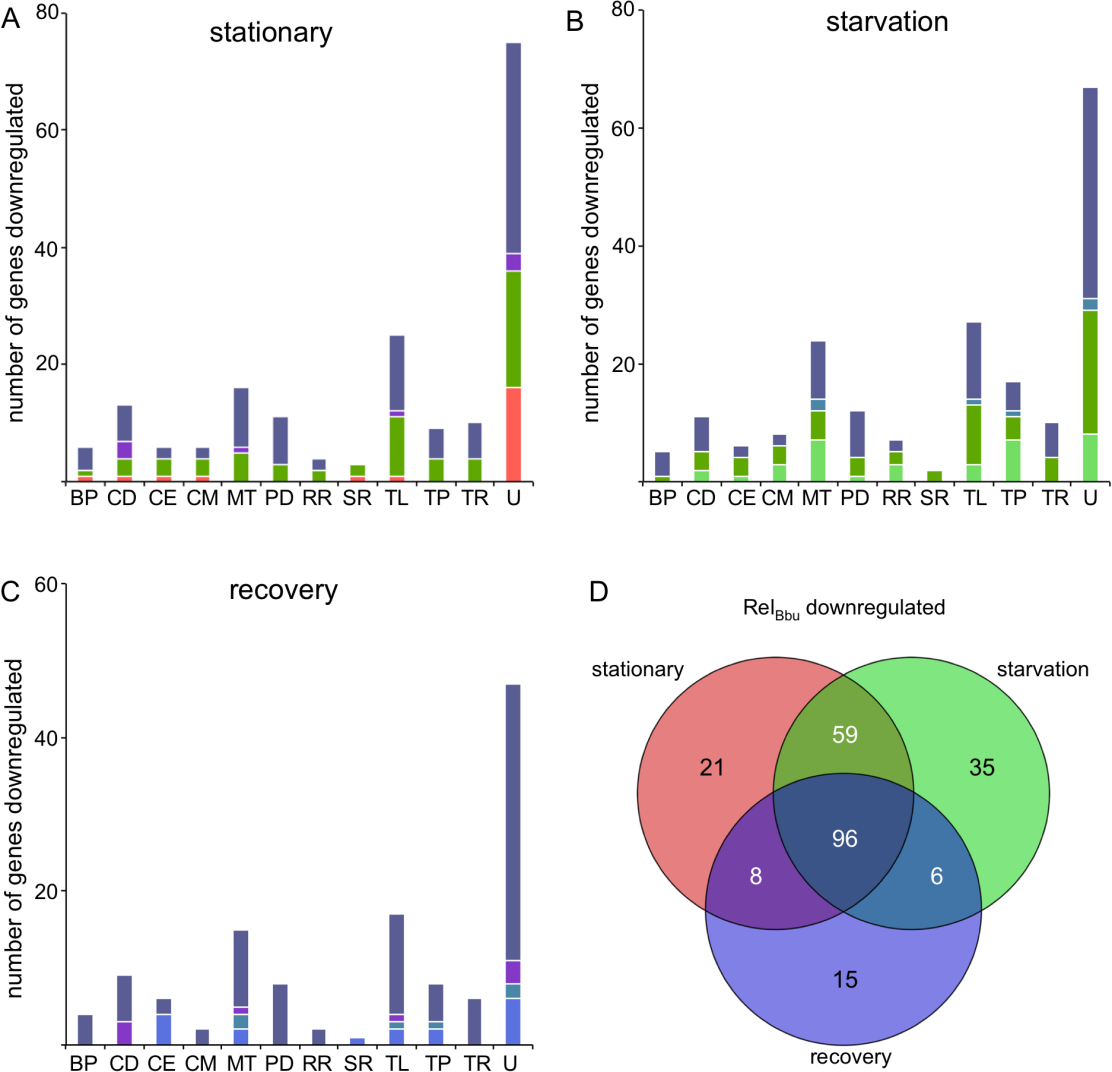
Rel_Bbu_-downregulated genes during nutrient stress. The number of genes significantly Rel_Bbu_-downregulated (lower in wild type than *rel*_Bbu_^-^) greater than twofold as determined by RNA-seq in (A) stationary phase, (B) starvation and (C) recovery from starvation. Bars are color-coded based on the color scheme assigned in (D) for unique and overlapping conditions that downregulate genes. (D) Venn diagram of the number of genes Rel_Bbu_-downregulated in stationary phase, starvation and recovery from starvation. Functional gene category abbreviations are the same as in Fig 8.

More genes are downregulated (higher expression in the *rel*_Bbu_ mutant than in wild type) than upregulated by Rel_Bbu_ under all conditions examined (Figs 9 and 10). Using the same parameters for significance and a twofold cutoff, 184 genes were repressed by Rel_Bbu_ in stationary phase, 196 genes during starvation, and 125 genes during recovery from starvation (Fig 10 and S8–S10 Tables). A higher percentage of the Rel_Bbu_-downregulated genes were common to all conditions (19%) than were common among Rel_Bbu_-upregulated genes (9%). In fact, all the bacteriophage (BP), cell motility (CM), protein degradation (PD), DNA replication and repair (RR), and transcription (TR) genes downregulated by Rel_Bbu_ during recovery were common to all three conditions (Fig 10C, gray bars). Similar to the Rel_Bbu_-upregulated genes, stationary phase and starvation shared the most similar set of downregulated genes (59 genes). After genes of unknown function, the categories with the most Rel_Bbu_-downregulated genes were metabolism and translation (TL) (Fig 10A–10C). While many of the repressed genes unique to stationary phase (Fig 10A, red bars) and recovery (Fig 10C, blue bars) are of unknown function, the repressed genes unique to starvation are mainly divided among metabolic, replication and recombination, and transport proteins (TP) (Fig 10B, light green bars). The majority of downregulated translation genes encode 50S and 30S ribosomal proteins, as well as translation initiation and elongation factors, whose regulation was shared between all three conditions (Fig 10 and S8–S10 Tables). Rel_Bbu_ also downregulated expression of the RNA polymerase subunits *rpoB* (*bb0389*), *rpoC* (*bb0388*) and *rpoD* (*bb0712*) in all conditions (S8–S10 Tables). Consequently, perhaps not surprisingly, Rel_Bbu_ represses expression of ribosomal subunits and RNA polymerase subunits to mediate cellular adaptation to nutrient stress. Rel_Bbu_ exerted similar control of the genes encoding proteins involved in transcriptional regulation: all of these genes that are repressed during recovery are common to all conditions, and stationary phase and starvation share all ten repressed transcriptional regulator genes (Fig 10A–10C, TR).

Most of the genes encoding known proteases and peptidases were repressed by Rel_Bbu_ (Fig 10A–10C and S8–S10 Tables). All of the protease genes repressed during recovery are common to all three conditions, while stationary phase and starvation share all protease genes but one (Fig 10A–10C, PD). These include the ATP-dependent proteases encoded by *clpP1* (*bb0611*), *clpX* (*bb0612*), *ftsH* (*bb0789*), and both *lon* paralogs (*bb0613* and *bb0253*), which are repressed by under all conditions (S8–S10 Tables). Additionally, the ATP-dependent proteases encoded by *htrA* (*bb0104*) and *hlsV* (*bb0296*) are repressed in stationary phase and starvation. The role of most proteases remains unknown in *B*. *burgdorferi*, but some are likely involved in protein quality control. *ftsH*, which is repressed in each condition, encodes a protease that, in *E*. *coli*, regulates phage λ life cycle by degrading the cII protein [85,86].

The group of contiguous genes encoding all the enzymes for the mevalonate biosynthetic pathway was also repressed by Rel_Bbu_. This appears to be the only route for biosynthesis of isoprenoids in *B*. *burgdorferi* [6,87]. During starvation, *hmgs*, *fni*, *hmgr*, *mvaD*, *pmk*, and *mvk* (*bb0683*-*bb0688*) were all repressed by Rel_Bbu_ (S9 Table), while *bb0683*-*bb0687* are repressed in recovery (S10 Table) and only *bb0685*-*bb0687* are repressed in stationary phase (S8 Table). Previous studies have shown that external acetate levels influence the mevalonate pathway and that transcript levels of most of the genes in this pathway are lower in *B*. *burgdorferi* in ticks than in dialysis membrane chambers (DMCs) in mice [84,87]. These data together with our results support a role for Rel_Bbu_ in the tick.

Many more genes on the cp32s were Rel_Bbu_-repressed (47 in stationary, 40 in starvation and 23 in recovery) than were Rel_Bbu_-induced (11 in stationary, 8 in starvation and 12 in recovery) (S5–S10 Tables). The majority of these genes encode hypothetical proteins of unknown function, but many are located on the putative late phage operons [88,89]. These data, along with the repression of *ftsH*, raise the possibility that Rel_Bbu_ regulates the *B*. *burgdorferi* prophages, a relationship that has been reported for λ [90,91].

## Discussion

*B*. *burgdorferi* must persist in an *Ixodes* tick in order in to traverse its enzootic cycle. Yet the molecular mechanisms by which the spirochete survives extreme nutrient limitations between blood meals taken by larval and nymphal ticks are for the most part unexplored. We now show that Rel_Bbu_, which controls (p)ppGpp levels in *B*. *burgdorferi*, is important for persistence in the tick vector. Furthermore, Rel_Bbu_ is the first factor shown to be required for *B*. *burgdorferi* survival specifically during starvation *in vitro*, conditions mimicking the midgut environment between blood meals in the flat tick. These findings stress the importance of (p)ppGpp not only as an integrator of environmental growth conditions and bacterial replication, but also as a direct signal for virulence factor expression [25,49,50].

### (p)ppGpp levels increase in response to nutrient limitation

We found that (p)ppGpp levels increased when *B*. *burgdorferi* were starved for nutrients (shifting from BSK + RS to RPMI) for 30 min and 6 h (Fig 1). Recovery from starvation returned (p)ppGpp levels to those measured in actively growing cells. The production of (p)ppGpp in response to nutrient stress was Rel_Bbu_-dependent. There have been conflicting reports regarding changes in (p)ppGpp levels in *B*. *burgdorferi* during nutrient limitation. Our data agree with the results of Concepcion et al. [63], but not Bugrysheva et al., who found that (p)ppGpp levels did not increase during starvation for serum, yeastolate or neopeptone [61]. This discrepancy is likely due to our study and Concepcion et al. [63] both starving cells in RPMI while the other report used BSK, which is based on the cell culture medium CMRL and contains bovine serum albumin (BSA) and rabbit serum. The presence of BSA and associated fatty acids, and other lipids, as well as other components of CMRL, may not induce the stringent response and (p)ppGpp production.

The specific signals that induce Rel_Bbu_-mediated (p)ppGpp accumulation in *B*. *burgdorferi* have not been identified. In many other bacteria, limiting amino acids activates RelA to synthesize (p)ppGpp, while the lack of other nutrients such as carbon, fatty acids, iron, and phosphate activate SpoT-mediated (p)ppGpp synthesis over hydrolysis. In bifunctional enzymes, like RSH and Rel, the synthetic/hydrolytic activities on the N-terminal region are coordinated by conformational changes and regulatory domains, such as ACT and TGS (threonyl tRNA synthetase, GTPase, SpoT/RelA), which are in the C-terminal region [25,29,92,93]. Starvation for FA is communicated via FA-bound acyl carrier protein to the TGS domain in SpoT proteins to favor (p)ppGpp synthesis [35]. Regulation of ppGpp levels by FA is an attractive hypothesis in *B*. *burgdorferi* since the spirochete lacks the ability for *de novo* FA synthesis [6,7] and Rel_Bbu_ contains a predicted C-terminal TGS domain. However, the function of the TGS domain of Rel_Bbu_ is not known, as this domain has so far only been implicated in sensing FA in SpoT proteins [94]. Additionally, the TGS domain mediates Rel_Mtb_ oligomerization as well as association with ribosomes/tRNA/mRNA in *Mycobacterium tuberculosis* [95,96]. Other candidates that may regulate Rel_Bbu_ activity include components present in BSK + RS, but not in RPMI, such as neopeptone, yeastolate, and possibly metals associated with serum, but a detailed description of the nutrients and domains targeted that control (p)ppGpp levels will require a molecular dissection of the Rel_Bbu_ enzyme and component analysis of extracellular medium.

Along with the increase in (p)ppGpp levels during starvation, we also observed an accumulation of PP_i_ and, predictably, a decrease in GTP levels. GTP is consumed by Rel_Bbu_ to synthesize pppGpp. Exactly how pyrophosphate levels increase is unclear, but one possibility is that the activity of the regulatory glycolytic enzyme pyrophosphate phosphofructokinase (PP_i_-PFK; BB0020) is decreased and less PP_i_ is used to form fructose 1,6-diphosphate. PP_i_-PFK activity is reversible [97], unlike ATP-PFK, so *B*. *burgdorferi* could be converting fructose 1,6-diphosphate to PP_i_ and fructose 6-phosphate, thus increasing PP_i_ levels [98]. In fact, accumulation of fructose 6-phosphate, a substrate of PFK, is a key regulator of the stress response during nutrient starvation via the universal stress protein in *E*. *coli* [99]. However, expression of *pp*_*i*_ *-pfk* (*bb0020*) was not affected in the *rel*_Bbu_ mutant, so any regulation by Rel_Bbu_ would likely be through a post-transcriptional mechanism. Rel_Bbu_ did repress expression of a second *pfk* gene, *bb0727* (S8–S10 Tables), although BB0727 lacks PP_i_-PFK activity and is thought to be an evolutionary link between PP_i_-PFK and ATP-PFK [100]. Illuminating the role of BB0727 and its potential effect on PP_i_ levels in the spirochete will require further investigation.

### Rel_Bbu_ controls RB formation during nutrient limitation

Since discovered almost two decades ago by Brorson and Brorson [57], the round body, or condensed cyst form, of *B*. *burgdorferi* has been largely ignored until recently. Although the physiological role of *B*. *burgdorferi* RBs remains unknown, it appears to be a morphological adaptation to environmental stress, particularly nutrient starvation [58,59]. While RBs represent an unusual spirochete morphology, they are not simply an *in vitro* culture artifact as they have been identified *in vivo* in tick midguts, are viable, and rapidly convert back to the distinctive flat-wave morphology of *B*. *burgdorferi* [57,60]. Our findings suggest that (p)ppGpp may be an important intracellular signal for RB formation during nutrient stress: strains unable to produce (p)ppGpp (*rel*_Bbu_ mutant) not only more frequently form RBs, but they also have disrupted membranes and are less viable (Figs 4 and 5). One advantage to forming RBs may be to decrease the spirochete’s surface area, thus better adapting *B*. *burgdorferi* to environmental oxidative and osmotic stresses, as well as possible evasion from the tick immune system. Remarkably, a spherical spirochete is not without precedent: Sphaerochaeta, a recently isolated free-living spirochete from freshwater sediment, has never been observed with a flat wave or helical morphology [101,102].

The molecular mechanisms controlling RB formation are unknown, but Dunham-Ems et al. showed that an *rpoS* null mutant formed RBs more frequently when starved for nutrients, but had no decrease in viability [60]. Our results that lack of Rel_Bbu_ increased RB formation and decreased survival during starvation, independent of alterations in *rpoS* transcript levels, suggest that (p)ppGpp influences the transition to RBs slightly differently than the RpoS-mediated pathway. In addition, there may be a connection between coenzyme A metabolism and RB formation: a coA-disulfide reductase (*cdr*, *bb0728*) mutant is more likely to form RBs than wild type during starvation [60] and we found that dephospho-CoA kinase (*coaE*) transcript levels decreased during starvation and increased during recovery in wild-type cells (S2 and S3 Tables).

### Rel_Bbu_ is necessary for *B*. *burgdorferi* tick persistence

Our data demonstrate that Rel_Bbu_, and presumably (p)ppGpp, are important for *B*. *burgdorferi* persistence in the tick vector, specifically between the fed larvae and fed nymph, and likely initiate a program to adapt to the nutrient-limited environment of the tick midgut. Previous studies have identified a number of other *B*. *burgdorferi* genes that differ in expression between *in vitro* conditions designed to mimic flat and fed ticks, and others important for *in vivo* tick persistence [3,13,84,103]. IF microscopy data suggest that *rel*_Bbu_ mutant strains do not survive the molt, as fewer spirochetes were seen in the midguts of flat nymphs infected with the mutant compared to those infected with the wild type, while qPCR data point to compromised survival of *rel*_Bbu_ mutants during the nymphal blood meal. One possible explanation for this discrepancy is that DNA from nonviable *B*. *burgdorferi* in flat nymphs is still detected. We hypothesize that 25% of mice can be infected by transmission from *rel*_Bbu_ mutant-infected nymphs (Table 1) due to decreased spirochete loads in the nymphs, but Rel_Bbu_ may play a role in transmission and host infection. In fact, expression of genes associated with virulence in the host is upregulated by Rel_Bbu_, but we found no qualitative differences in mouse infectivity.

Rel_Bbu_-dependent tick persistence is likely due, at least in part, to upregulation of the *glp* operon: *glpF*, *glpK* and *bb0242* (which encodes a hypothetical protein) are Rel_Bbu_-upregulated during starvation (S6 Table), and *glpF* and *bb0242* are Rel_Bbu_-upregulated during stationary phase (S5 Table). Furthermore, *glpF* is upregulated in wild-type cells during starvation compared to stationary phase (S1 Table) and downregulated, along with *glpK*, in recovery compared to starvation (S4 Table). Previous studies have shown that glycerol and the *glp* operon, which mediates glycerol uptake and metabolism, are important for tick persistence [19,20]. This operon is induced by glycerol and temperature, and in both larvae and nymphs compared to mammalian adapted *B*. *burgdorferi* in DMCs [19,84,104]. Moreover, the *glp* operon is upregulated by the intracellular second messenger c-di-GMP [20,105], which has been implicated in the virulence of many pathogens [106,107] as well as in the persistence of *B*. *burgdorferi* in the tick [20–23,108–110]. Microarray analysis has revealed that c-di-GMP is a global transcriptional regulator affecting many genes, including the RpoS regulon, through the c-di-GMP-binding protein PlzA [20,24,105,108,111]. Comparison of the (p)ppGpp and c-di-GMP regulons provides new insights into subtle changes of the transcriptional landscape. For example, (p)ppGpp and c-di-GMP both induce expression of *bb0240*-*bb0242* of the *glp* operon (S5 and S6 Tables), but (p)ppGpp represses *glpD* (*bb0243*) during stationary phase and recovery (S8 and S10 Tables; S4 Fig), while c-di-GMP upregulates this gene [20,105]. GlpD is predicted to convert glycerol-3-P to dihydroxyacetone-P, a reaction directing glycerol-3-P to glycolysis [6,7]. Therefore, c-di-GMP may favor glycerol utilization for glycolysis while (p)ppGpp-mediated repression of *glpD* may direct glycerol to a different fate, such as phospholipid and lipoprotein biosynthesis [6,7]. During preparation of this manuscript, Bugrysheva et al. published a description of the Rel_Bbu_ transcriptome [83]; there are many differences between this study and ours, notably they used 1) a mutant that is not infectious in mice [62], and is likely missing plasmid components of the genome, 2) an oligonucleotide microarray, and 3) growth conditions that do not alter (p)ppGpp levels [61]. However, they did also find that Rel_Bbu_ upregulates expression of the *glp* genes [83], although our data suggest that the *glpD* gene of this operon is differentially regulated as seen in a plot of the RNA-seq reads mapped to this region (S4 Fig). Previous work has also suggested that *glpD* expression follows that of *glpF* and *glpK* [20,104,105,112]. Further investigation will be needed to resolve these discrepancies concerning *glpD* regulation.

Since both the enzymes that synthesize (p)ppGpp and c-di-GMP, Rel_Bbu_ and Rrp1, respectively, are important for *B*. *burgdorferi* survival in the tick and induce the *glp* operon, their levels and downstream effects are likely coordinated [26]. c-di-GMP levels in *B*. *burgdorferi* are regulated by three enzymes [109]: the response regulator Rrp1 is a diguanylate cyclase [105,113] that combines two molecules of GTP to form c-di-GMP and two molecules of PP_i_, while two phosphodiesterases, PdeA [114] and PdeB [23], hydrolyze c-di-GMP to yield two molecules of GMP. As previously discussed, Rel_Bbu_ is responsible for both synthesis and hydrolysis of (p)ppGpp: synthesis transfers PP_i_ from ATP to GDP or GTP to yield ppGpp and pppGpp, respectively, and AMP; hydrolysis produces GDP or GTP and PP_i_. While there is no known enzyme directly linking (p)ppGpp and c-di-GMP, production of both second messengers consumes GTP (in the case of pppGpp) while PP_i_ is a product of c-di-GMP synthesis and (p)ppGpp hydrolysis; thus, the two pathways could influence each other by affecting the concentration of substrates or products. In fact, (p)ppGpp and c-di-GMP recently were reported to have overlapping functions in *Mycobacterium smegmatis* [115].

The two nucleotide messengers may also coordinate their effects by targeting expression of the transcription factor BosR. BosR was identified as the *Borrelia* oxidative stress regulator [79,116] and more recently as an important global transcriptional activator of virulence gene expression mediated by the dual sigma factor (RpoN-RpoS) regulatory pathway [80,81,117,118]. While previous microarray studies did not find *bosR* transcript significantly upregulated by Rrp1 [20,105], more recent work reported that c-di-GMP upregulates *bosR* transcriptionally and post-transcriptionally via the c-di-GMP-binding protein PlzA [111,119]. We found that Rel_Bbu_ ((p)ppGpp) upregulates *bosR* expression (S5 and S6 Tables), suggesting that Rel_Bbu_ may have a dominant effect over Rrp1 on *bosR* transcript levels, thus offering an explanation for the differential regulation observed in *rrp1* and *plzA* mutants. The Rel_Bbu_-dependent increase in *bosR* during stationary phase, but not in starvation, coincided with increased *rpoS* expression (S5 Table). This difference could be explained by the phosphorylation state of Rrp2, a response regulator that is required for *rpoS* expression [120,121]. In a previous study, the expression of *bosR* was not identified as Rel_Bbu_-dependent [83]. The mechanism(s) of (p)ppGpp and c-di-GMP coordination remains mysterious, but it appears not to be directly transcriptional as *rrp1* and *rel*_Bbu_ mutants do not affect each other’s transcript levels (S5–S10 Tables) [20,105]. Further studies are needed to elucidate the network of interactions between these two intracellular messengers.

### Nutrient stress and the Rel_Bbu_ ((p)ppGpp) regulon

Rel_Bbu_-mediated mechanisms both activate and repress the expression of numerous genes during nutrient stress, with the suites of genes targeted being more similar during stationary phase and starvation compared to recovery from starvation. Our finding that Rel_Bbu_ mediates changes in gene expression in the absence of starvation (low or no (p)ppGpp production; Fig 1) indicates that proper transcriptional regulation may have an absolute requirement for (p)ppGpp. The complete absence of (p)ppGpp may alter the balance of sigma factor use by RNAP, enhancing the sensitivity of some genes more than others to (p)ppGpp regulation. Alternatively, Rel_Bbu_-mediated transcriptional effects may be independent of (p)ppGpp and instead due to other as yet undefined functions of Rel_Bbu_. Determining the significance of decreased gene expression from the plethora of plasmids in the *rel*_Bbu_ mutant compared to the wild type (Rel_Bbu_-upregulated genes) must be carefully considered as these replicons can be lost during *in vitro* cultivation and transformation [122,123]. If this occurred, then all the genes on a given plasmid would appear to be repressed in the *rel*_Bbu_ mutant (Rel_Bbu_-upregulated). This was not the case in our data as each plasmid, including all of the cp32s, contained genes that were upregulated, repressed and not significantly changed under at least one condition (S5–S10 Tables). However, there remains the possibility that a small percentage of the cells in the population have lost a plasmid, thus slightly skewing the regulatory effect on the transcriptome. This concern is not relevant to genes on the chromosome, which cannot be lost in viable cells, or for genes whose expression is higher in the *rel*_Bbu_ mutant than in the wild type (Rel_Bbu_-repressed) as the spirochete does not typically gain plasmids.

A number of genes upregulated by Rel_Bbu_ during stationary phase and/or starvation encode adhesins whose products bind to the extracellular matrix of the host, such as *erpX* [124], *revA* [125], *bbk32* [126], and *bba33* [127], and are important for host infection [128–130]. Two adhesin genes, encoding decorin-binding proteins A (*dbpA*) and B (*dbpB*) [131], were induced by Rel_Bbu_ during starvation, but not in stationary phase (S5 and S6 Tables). Binding of DbpA and DbpB to decorin is important for infection and dissemination in the host [132–134]. A low-nutrient environment may seem at odds with host infection, but the extracellular space can be nutritionally inhospitable. For example, the articular cartilage of synovial joints is a smooth connective tissue containing decorin, and this extracellular space is not well vascularized and low in nutrients [135]. Therefore, as *B*. *burgdorferi* migrates to the synovial joint, the spirochete could encounter a nutrient-poor environment that signals Rel_Bbu_ to increase (p)ppGpp levels leading to the expression of *dbpBA* and binding to decorin, facilitating immune evasion and/or adhesion. Therefore, (p)ppGpp may provide the transcriptional regulation that differentiates *dbpBA* expression from the expression of other genes, such as *ospC*, governed by the RpoN-RpoS pathway [64,112,136–138].

Evasion of the host immune system during *B*. *burgdorferi* infection is accomplished, at least in part, by antigenic variation of the surface lipoprotein VlsE [122]. The epitope diversity is generated when a portion of the *vlsE* gene (the expression locus) is replaced by a silent *vls* cassette via gene conversion [77,78,139]. The mechanism of *vlsE* induction during infection remains unknown, but our transcriptome analysis showed that Rel_Bbu_ upregulates the expression locus *vlsE* (*bbf0041*) under all conditions tested (S5–S7 Tables). In addition, *vlsE* was significantly upregulated in wild-type cells recovering from starvation (S3 Table) suggesting nutrient availability may be a signal for expression. Other factors that induce *vlsE* expression *in vitro* include oxygen tension [140], pH [141], AI-2 [142], and mammalian epithelial cells [143]. Unexpectedly, Rel_Bbu_ repressed expression of the “silent” *vls* cassettes. This result was somewhat surprising considering that these genetic elements have been considered to be transcriptionally inert. While the observed increase in *vls* cassettes expression is significant, the level of expression in the *rel*_Bbu_ mutant is modest compared to *vlsE* (S5 Fig). Rel_Bbu_-mediated repression of the *vls* cassettes coupled with upregulation of the *vlsE* expression site may represent a mechanism to ensure that only the variable *vls* cassettes inserted into the expression locus are transcribed. Sequencing of the *vlsE* locus from the *rel*_Bbu_ mutant grown *in vitro* showed no difference in antigenic switching compared to wild type (6/6 *rel*_Bbu_ mutant clones had the same *vlsE* sequence as the wild-type parental strain).

Rel_Bbu_-mediated repression of another intriguing gene, *cgtA* (*bb0781*), was seen during all three conditions (Fig 10 and S8–S10 Tables). CgtA is a small GTPase of the Obg family, which, in *Vibrio cholerae*, influences many cellular functions including repression of the stringent response, possibly by its interaction with SpoT [144]. CgtA has been implicated in numerous cellular processes including sporulation, DNA repair, and ribosome assembly via interactions with the 50S ribosomal subunit [145]. We found that almost half of the genes for 50S ribosomal subunits were also repressed by Rel_Bbu_ during starvation (S9 Table), raising the possibility that repression of some 50S ribosomal genes and *cgtA* may be part of the mechanism to inhibit translation during the stringent response. Furthermore, if CgtA modulates the stringent response [146] by increasing ppGpp hydrolysis [144], then Rel_Bbu_-mediated repression of *cgtA* could be a positive feedback during the stringent response: *cgtA* is repressed as (p)ppGpp levels increase and (p)ppGpp is less likely to be hydrolyzed, thus accelerating (p)ppGpp accumulation. The converse would occur during recovery from the stringent response as (p)ppGpp is hydrolyzed.

### cp32s and prophage induction

While the cp32s of *B*. *burgdorferi* are extensively homologous, we were able to distinguish paralog-specific transcripts using RNA-seq (see Materials and Methods). Rel_Bbu_ repressed 110 cp32 genes (S8–S10 Tables) and induced 31 cp32 genes (S5–S7 Tables) under all conditions examined. The cp32s, or at least some members, are lysogenic prophages of the bacteriophage ϕBB-1 [89,147] that can transduce between strains [122,148]. Induction of ϕBB-1 upregulates 30 genes that constitute a late operon from *bbl42* to *bbl28* (using the paralog designations from cp32-8) [88]. *blyA*, one of the induced paralogous genes located on the cp32s, as well as other plasmids, encodes a holin predicted to be required for phage release [149]. In *E*. *coli*, (p)ppGpp controls the λ lysis-lysogeny decision via transcriptional regulation [91]: moderate levels of ppGpp maintain lysogeny [150,151]. This is consistent with our observations that the *rel*_Bbu_ mutation induces prophage gene expression in *B*. *burgdorferi*, possibly via the FtsH protease, which promotes λ lysis in *E*. *coli* [85,86]. We hypothesize that Rel_Bbu_ regulates prophage development in *B*. *burgdorferi*, but further studies are required to probe how our observed changes in gene expression relate to lysis, lysogeny and transduction.

### Summary

We have found that Rel_Bbu_ is necessary for *B*. *burgdorferi* persistence in the tick in the tick-murine model of Lyme disease. The Rel_Bbu_-produced (p)ppGpp is a global regulator of the genetic programs engaged during nutrient limitations in the tick that link morphology, metabolism and survival during this heretofore insufficiently studied phase of the enzootic cycle.

## Materials and Methods

### Ethics statement

All experiments involving the use of animals were approved by the University of Montana Institutional Animal Care and Use Committee (Animal Use Protocol # 041-11SSDBS) and in full compliance with the *Guide for the Care and Use of Laboratory Animals* from the National Institutes of Health.

### Bacterial strains and culture conditions

Low-passage *B*. *burgdorferi* strain B31-5A4 [152] (a gift from George Chaconas) and all mutant strains were maintained in Barbour-Stoenner-Kelly II (BSK) liquid medium, pH 7.6, containing 6% rabbit serum (RS) (Pel-Freez Biologicals) [153] without gelatin [154]. Cultures were inoculated at 1 × 10^5^ and grown at 23°C until late log phase (5 to 9 × 10^7^ cells ml^-1^) or inoculated at 1 × 10^3^ or 1 × 10^4^ cells ml^-1^ and grown at 35°C to late log or stationary phase (1 to 3 × 10^8^ cells ml^-1^) in BSK (pH 7.6 or 6.8) + RS. Cell density was determined using a Petroff-Hausser counting chamber [154]. *B*. *burgdorferi* strains were starved by centrifuging cultures at 9,000 × *g* for 5 min at room temperature (RT). Pellets were resuspended in RPMI 1640 without L-glutamine (Mediatech, Inc.) and without serum for the times indicated at 35°C.

To quantify the number of live *B*. *burgdorferi* cells from *in vitro* cultures, strains grown in BSK + RS to late log phase were divided into two separate cultures and one was starved in RPMI (as described above) and the other kept in BSK + RS. At the times indicated, equal volumes of each culture were plated in semi-solid BSK + RS and grown at 37°C in an incubator with 5% CO_2_ as previously described [154]. After two weeks, colonies were enumerated. Each value is the mean ± SEM from at least three independent experiments.

### Mutant construction

In order to generate a *rel*_Bbu_^-^ strain, a region of the chromosome upstream of the *rel*_Bbu_ gene was amplified by PCR using KOD polymerase (Novagen) with the primers rsh U866F and rsh 142R+Aat+Age and a region downstream of the *rel*_Bbu_ gene amplified using primers rsh 1939F+AatII and rsh D3102R+AgeI (S11 Table). PCR products were cloned into pCR2.1-TOPO (Invitrogen) and verified by DNA sequencing at the University of Montana Murdock Sequencing Facility. The upstream and downstream pieces were digested with AatII and AgeI and ligated together leaving a synthetic AatII site. The streptomycin/spectinomycin resistant cassette with the *flgB* promoter from *B*. *burgdorferi* [68] and *trpL* terminator from *Bacillus subtilis* [155] (*flgBp*-*aadA*-*trpLt*) was then inserted into the AatII site. The resulting plasmid was linearized by digestion with AhdI and ethanol-precipitated. Competent *B*. *burgdorferi* strain B31-5A4 was electroporated with 10 μg of linearized DNA as previously described [154] and transformed cells dilution plated in liquid BSK + RS [156] containing 50 μg ml^-1^ streptomycin in 96-well plates. Positive colonies were confirmed to have the *rel*_Bbu_ gene replaced by the *aadA* cassette by PCR analysis. Two different strains were constructed to complement the *rel*_Bbu_^-^ strain. The *rel*_Bbu_ ORF was PCR-amplified using the oligonucleotides rsh 1F+NdeI and rsh 2004R+AatII and cloned into pCR2.1-TOPO. The inducible *flac* promoter [69] (containing synthetic 3′ NdeI and 5′ AatII sites) was inserted upstream of the *rel*_Bbu_ ORF. The *flacp*-*rel*_Bbu_ construct was then inserted into the AatII site of pBSV2 [70] containing the *lacI* gene under control of the *B*. *burgdorferi flgB* promoter in the MCS, as previously described [157], to produce the construct pBS-*flacp*-*rel*_Bbu_. In the second construct to complement the *rel*_Bbu_^-^ strain, the *rel*_Bbu_ ORF and 365 nucleotides upstream containing the native promoter [61] were PCR-amplified using primers rsh U365+AatII and rsh 2004+AatII, cloned into pCR2.1-TOPO, subcloned into the AatII site of pBSV2 to generate pBS-*rel*_Bbu_, and verified by DNA sequencing. 10 μg of either pBS-*flacp*-*rel*_Bbu_ or pBS-*rel*_Bbu_ was used to transform the competent *rel*_Bbu_^-^ strain and transformants selected in 200 μg ml^-1^ kanamycin and 50 μg ml^-1^ streptomycin as described above.

### RNA isolation and qRT-PCR analysis

RNA was isolated and qRT-PCR performed as previously described [158]. Briefly, RNA was isolated from 50-ml cultures of *B*. *burgdorferi* grown at 23°C or 35°C under the conditions described for individual experiments using TRIzol (Invitrogen). Contaminating DNA was removed by treating samples with Turbo DNase (Ambion) followed by phenol/chloroform extraction. To ensure no DNA remained, samples were checked by PCR using the primers flaB 423F and flaB 542R before synthesizing cDNA. The SuperScript III kit (Invitrogen) was used to convert 1 μg of RNA to cDNA according to the manufacturer’s instructions. Primers and FAM-TAMRA labeled probes were designed using Primer Express 3.0 (Applied Biosystems) or MacVector (MacVector, Inc.). TaqMan qRT- PCR was performed in 96-well plates using TaqMan Universal PCR Master mix (Applied Biosystems) with an Applied Biosystems 7300 Real-time PCR thermo cycler and cycling conditions: 50°C 2 min; 95°C 10 min; 95°C 15 sec and 60°C 1 min for 40 cycles. Transcript amounts of *rel*_Bbu_ and *flaB* were calculated using a standard curve generated using known amounts of the *rel*_Bbu_ ORF or a portion of the *flaB* ORF (nucleotides 278–551 of the ORF) cloned into pCR2.1-TOPO, respectively. *B*. *burgdorferi* genomic DNA was used to generate standard curves for all other target sequences. Transcript copy number of the gene of interest in each sample (in triplicate) was determined using the internal standard curve and then normalized to the number of *flaB* copies. Each value is the mean ± SEM from at least three independent experiments.

### Fluorescence microscopy of WGA-Alexa Fluor 594 and propidium iodide-stained *B*. *burgdorferi*

*B*. *burgdorferi* cultures were pelleted by centrifuging at 10,600 × *g* for 5 min at RT. Cells were washed with 1.0 ml Dulbecco’s phosphate buffered saline (138 mM NaCl, 2.7 mM KCl, 8.1 mM Na_2_HPO_4_, and 1.5 mM KH_2_PO_4_; dPBS) and centrifuged again at 10,600 × *g* for 5 min at RT. Cells were resuspended in 100 μl dPBS and 1 μl wheat germ agglutinin (WGA)-Alexa Fluor 594 (1 mg ml^-1^ in dPBS + 5 mM MgCl_2_) (Molecular Probes) was added and gently mixed. After incubation at 37°C for 5 min in the dark, cells were collected by centrifuging at 10,600 × *g* for 5 min at RT, resuspended in 10 μl dPBS and wet mounted on slides. Slides were examined using an Olympus BX51 fluorescence microscope with 100x/1.30 NA or 40x/0.75 NA objectives. Images were processed using ImageJ (National Institutes of Health; http://rsbweb.nih.gov/ij/) and Pixelmator (Pixelmator Team, Ltd).

To determine which *B*. *burgdorferi* were dead following incubation in starvation media (RPMI without serum), cultures were collected by centrifugation at 10,600 × *g* for 5 min at RT and the cell pellet resuspended in 10 μl 0.85% NaCl. Propidium iodide (15 μM) was added to a final concentration of 1.5 μM and cells incubated for 15 min at RT in the dark. Samples were wet mounted on slides and examined by fluorescence microscopy as described above. The percentage of live cells under each condition for each strain was calculated as follows: 100 –((number of PI stained cells/total number of cells) x 100). Each value is the mean ± SEM from at least three independent experiments.

### Immunofluorescence microscopy of *B*. *burgdorferi*-infected ticks

*B*. *burgdorferi* persistence in ticks was assayed by IF microscopy using anti-*B*. *burgdorferi* antibodies as previously described [159,160]. Six ticks were crushed on a single slide and midguts separated in 10 μl of dPBS with 5 mM MgCl_2_ using 27-guage needles on silane-coated slides (LabScientific, Inc.). Midguts were air-dried for 30 min before being fixed in acetone for 10 min. Slides were washed 3 × 10 min in wash buffer (dPBS + 5 mM MgCl_2_ + 1% goat serum (Gibco, Life Technologies)) and then incubated with rabbit polyclonal anti-*B*. *burgdorferi* antibodies (a gift from Tom Schwan) at 1:200 dilution for 1 h. Slides were washed again as above and the primary antibodies detected using goat anti-rabbit Alexa Fluor 488 antibodies (Molecular Probes) at 1:500 dilution for 1 h. Slides were washed 2 × 10 min in wash buffer. Tick cells were stained by incubating slides for 5 min with WGA-Alexa Fluor 594 at 1:200 dilution in wash buffer. Slides were then washed a final time for 5 min. Coverslips were mounted on slides with ProLong Gold (Molecular Probes) and sealed with Permount (Fisher Scientific) and allowed to dry prior to examination by fluorescence microscopy as described above.

### Quantification of *B*. *burgdorferi* in ticks by qPCR analysis

Persistence of *B*. *burgdorferi* in ticks was quantified by isolating DNA from fed larvae (one week post feeding; groups of 5), flat nymphs and fed nymphs (one week post feeding) by grinding with a pestle in a 1.5-ml tube and using the DNeasy Blood/Tissue kit (Qiagen) [161]. TaqMan qPCR was done as described above using the primers and probe to the *flaB* gene listed in S11 Table. Values are expressed as the number of spirochetes/tick based on the genome equivalents where one copy of the *flaB* gene = one genome = one spirochete.

### Scanning electron microscopy

*B*. *burgdorferi* cultures were collected by centrifugation at 10,000 × *g* for 10 min at 4°C, the cell pellet resuspended in fixative (20 mM sodium cacodylate, pH 6 with 2.5% v/v glutaraldehyde) and cells were fixed overnight at 4°C. Cells were then centrifuged again, washed once in ddH_2_O and fixed in 2% osmium tetroxide for 2 h at 4°C. Cell pellets were washed twice in ddH_2_O, resuspended in ddH_2_O and the cells were loaded onto a 0.6 μm filter using a 1-ml syringe. Cells were gently dehydrated for 10 min each in a graded ethanol series; 35%, 50%, 70%, 90%, 95%, and twice in 100% EtOH using a 1-ml syringe. The filter was removed after the final 100% EtOH wash and placed in 100% hexamethyldisilazane for 30 min. The filter was air dried and placed on an adhesive carbon tab on a 13 mm aluminum stub. Filters with bacteria were coated with gold and palladium in a Pelco Model 3 sputter coater for 30 sec. After coating, samples were imaged in a Hitachi S-4700 Field Emission scanning electron microscope.

### The tick-mouse model of infection

To determine the murine infectivity of *B*. *burgdorferi* strains, C3H-HeJ female mice were intradermally needle-inoculated in the hind leg with either 1 x 10^5^ or 1 × 10^6^ cells of wild-type (B31-5A4), *rel*_Bbu_^*-*^ or complemented strains [162]. Three weeks after inoculation, mouse ear tissue was collected and cultured in BSK + RS containing 50 μg ml^-1^ rifampicin, 20 μg ml^-1^ phosphomycin and 2.5 μg ml^-1^ amphotericin B. Cultures were screened for *B*. *burgdorferi* growth by dark-field microscopy. To examine strains for dissemination, mice were sacrificed five weeks post inoculation and ear, ankle and bladder tissues collected, cultured and screened for *B*. *burgdorferi*.

To examine tick acquisition of *B*. *burgdorferi* strains, naïve *Ixodes scapularis* larvae (National Tick Research and Education Resource, Oklahoma State University) were allowed to feed to repletion on infected mice. Fed larvae were collected and kept in a bell jar containing a saturated solution of K_2_SO_4_ to generate a 98% humidified atmosphere. One week after collection, fed larvae were assayed for *B*. *burgdorferi* by IF microscopy and the number of bacterium per tick were quantified by qPCR as described above. Flat and fed nymphs (one week post repletion) were analyzed for *B*. *burgdorferi* by IF microscopy and qPCR to quantify persistence. To test strain transmission from ticks to mice, five flat, infected nymphs were placed on each naive C3H-HeJ female mouse (at least three mice per *B*. *burgdorferi* strain) and allowed to feed to repletion. Mice were screened for *Borrelia* infection by culturing tissues collected three and five weeks after infestation, as described above.

### Measurement of (p)ppGpp

*B*. *burgdorferi* was grown to ~5 x 10^6^ cells ml^-1^ and 500 μl collected by centrifugation (8,600 × *g* for 5 min at RT), resuspended in the same volume of phosphate-free BSK + RS and grown for 12 h before addition of 20 μCi ml^-1 32^P orthophosphate (PerkinElmer). Cells were labeled for 24 h and 500 μl culture samples were collected by centrifugation at 9,000 × *g* for 7 min at RT. Cell pellets were resuspended in BSK + RS or RPMI containing 20 μCi ml^-1 32^P orthophosphate and incubated at 35°C for the indicated times. Samples were collected by centrifugation at 12,000 × *g* at 4°C for 5 min. Cell pellets were rinsed with 50 μl cold dPBS. Cells were lysed and nucleotides extracted by addition of 30 μl of cold 6.5 M formic acid (Fisher Scientific). Samples were incubated on ice for 10 min and stored at -80°C. Cell debris was pelleted by centrifugation (20,800 × *g*, 5 min at 4°C) before separation by thin layer chromatography (TLC). Polyethylenimine (PEI) cellulose TLC plates (EMD) [163] were pre-run in ddH_2_O to remove impurities and dried before 8 μl of each sample was spotted on the plate and allowed to dry. Samples were resolved in 1.5 M KH_2_PO_4_, pH 3.4. Plates were dried, covered with plastic and exposed to an intensifying screen for 48–72 h. Screens were analyzed using a Fujifilm FLA-3000G Phosphorimager. Three independent experiments were performed and pppGpp, ppGpp and GTP levels quantified by densitometry using ImageGauge. Values represent (p)ppGpp / (p)ppGpp + GTP and error bars are SEM [164]. Statistical significance (*P* < 0.05) was determined by one-way ANOVA with a Tukey’s *post-hoc* test.

### RNA sequencing

Total RNA was isolated using a hot phenol protocol [165]. Total RNA was treated with DNase I (Roche) following the manufacturer′s protocol. RNA integrity was measured using the Agilent 2100 Bioanalyzer. RNA with an RNA Integrity Number (RIN) above 9.0 was used for cDNA library construction. Directional (strand-specific) RNA-seq cDNA libraries were constructed with a ligation-based protocol as previously described [166] except a different 5′ end RNA linker (5′-ACACUCUUUCCCUACACGACGCUCUUCCGAUCU-3′) and corresponding forward primer for PCR (5′-AATGATACGGCGACCACCGAGATCTACACTCTTTCCCTACACGACGCTCTTCCGATCT-3′) were used. Total RNA was depleted of rRNA using the Ribo-Zero RNA removal kit for gram-negative bacteria (Epicenter). 250 ng of RNA was fragmented using the RNA fragmentation reagents (Ambion) per the manufacturers protocol at 70°C for 5 min. RNA was treated sequentially with tobacco acid phosphatase (Epicenter) and calf intestinal phosphatase (New England Biolabs) to remove 5′-end phosphates. Finally, RNA was treated with polynucleotide kinase (T4 PNK; New England Biolabs) without ATP to remove 2′-3′ cyclic phosphates for 4 h at 37°C per the manufacturer’s protocol. A 3′-end adaptor, based on the Illumina multiplexing adapter sequence (oligonucleotide sequences 2007–2014 Illumina, Inc. all rights reserved) blocked at the 3′ end with an inverted dT, was phosphorylated at the 5’ end using T4 PNK (New England Biolabs) per the manufacturer’s protocol. The 3′ multiplex adapter was ligated to the 3′ ends of the RNA using T4 RNA ligase (New England Biolabs) at 20°C for 6 h following the manufacturer’s protocol. RNA was size-selected (75–300 nt) and purified over a denaturing 8% polyacrylamide/8M Urea/TBE gel. The 5′ ends were phosphorylated with T4 PNK (New England Biolabs) following the manufacturer’s protocol. The Illumina 5′ end adapter was ligated to the 5′ ends using T4 RNA ligase (New England Biolabs). The ligated RNAs were size selected (100–400 nt) and gel purified as described above. The di-tagged RNA libraries were reverse-transcribed with SuperscriptII reverse transcriptase (Invitrogen) using random nonomers per the manufacturer’s protocol. cDNA libraries were prepared from wild-type, *rel*_Bbu_^*-*^ and complemented strains at three different time points and from two biological replicates (for a total of 3 x 3 x 2 = 18 samples) and were sequenced on an Illumina HiSeq 2000 with single-end 50-base-pair reads at the Campus Science Support Facilities Next Generation Sequencing unit (http://www.csf.ac.at/facilities/next-generation-sequencing/).

The reads were demultiplexed and adapters were clipped with cutadapt. After quality control, the reads (between 31–51 Mio reads per sample) were mapped to the *B*. *burgdorferi* B31 reference genome (GenBank Ids: AE000783, AE001583, AE000793, AE001582, AE000785, AE000794, AE000786, AE000784, AE000789, AE000788, AE000787, AE000790, AE001584, AE000791, AE000792, AE001575, AE001576, AE001577, AE001578, AE001579, AE001580, and AE001581) with NextGenMap 0.4.10 [167] using standard parameters and a minimum identity threshold of 90%; multireads (reads with mapping equally well to more than one location on the genome) were pruned. NextGenMap mapped between 69% and 86% of the reads with a mapping quality larger than 20. This corresponded to 26–41 Mio reads per dataset or a theoretical genome-wide coverage of 855-1368X. FeatureCounts [168] was used to calculate read counts for all datasets. We considered only genes present in the Schutzer annotation set [169], thus ignoring all reads that map to tRNAs (between 91% and 95% of the mapped reads). The final average coverage per gene was between 74X and 233X for the different datasets (S12 Table). From the read counts, we calculated differential expression between various conditions/time points using edgeR and DESeq and filtered the results by adjusted *P*-value ≤ 0.05. *P*-values were adjusted using Benjamini and Hochberg’s algorithm to control the false discovery rate. We observed very little variance between our biological replicates which, in turn, resulted in small differences between conditions being assigned very low *P*-values (i.e., a large number of genes were called “significantly differentially expressed” by edgeR). For this reason, we further filtered the list of DE genes by log-fold change (LFC) and considered only genes with a difference in normalized read counts (ABS (LFC) ≥ 1, which corresponds to an estimated twofold expression increase or decrease). We also extracted strand-specific, normalized depth-of-coverage signals (i.e., the counts of reads overlapping a particular genomic position) from the read alignments using CODOC [170] and converted them to the BigWig data format to enable manual inspection in a genome browser.

## Supporting Information

S1 Fig PP_i_ and GTP separated by PEI cellulose TLC. ^32^PP_i_ and α-^32^P GTP were separated by TLC, plates were dried, exposed to a phosphor screen and visualized using a phosphorimager.
(TIFF)

S2 Fig WGA-Alexa Fluor 594 staining of *B*. *burgdorferi*. Wild-type *B*. *burgdorferi* grown in BSK + RS were incubated with WGA-Alexa Fluor 594 and visualized by DIC (A) and fluorescence microscopy (B).
(TIFF)

S3 Fig Comparison of Rel_Bbu_-mediated changes in gene expression between RNA seq and qRT-PCR. Fold changes represent transcript levels (wild type/*rel*_*Bbu*_ mutant). Values for RNA seq are from S5–S10 Tables. TaqMan qRT-PCR was used for independent measurements of transcripts as described in the Materials and Methods for cultures grown to stationary phase, during starvation and recovery from starvation. qRT-PCR transcript levels were normalized to *flaB* levels. Values are the average of at least two independent experiments. The primers and probes used to measure transcript levels of *dbpB*, *vlsE*, *cgtA*, *glpF*, *chbA* and *gidA* are listed in S11 Table.
(TIFF)

S4 Fig RNA-seq reads mapped to *glp* operon in wild type and *rel*_Bbu_^-^. The RNA-seq results displayed in a coverage map of libraries in the wild-type and *rel*_Bbu_^-^ mutant strains at stationary phase and starvation for the four genes of the *glp* operon. The height at each position indicates the number of reads that mapped to that base, with the highest read being 172. The genome context is depicted below the coverage maps.
(TIFF)

S5 Fig RNA-seq reads mapped to *vlsE* and *vls* cassettes in wild type and *rel*_Bbu_^-^. The RNA-seq results for (A) *vlsE* and (B) *vls* “silent” cassettes (*bbf32*) displayed in a coverage map of libraries in the wild-type and *rel*_Bbu_^-^ mutant strains at stationary phase. The height at each position indicates the number of reads that mapped to that base, with the highest read being 82 in (A) and 7 in (B). The genome context is depicted below the coverage maps.
(TIFF)

S1 Table Genes upregulated in wild-type *B*. *burgdorferi* during starvation. (XLSX)

S2 Table Genes downregulated in wild-type *B*. *burgdorferi* during starvation. (XLSX)

S3 Table Genes upregulated in wild-type *B*. *burgdorferi* during recovery from starvation. (XLSX)

S4 Table Genes downregulated in wild-type *B*. *burgdorferi* during recovery from starvation (XLSX)

S5 Table Genes upregulated by Rel_Bbu_ in stationary phase. (XLSX)

S6 Table Genes upregulated by Rel_Bbu_ during starvation. (XLSX)

S7 Table Genes upregulated by Rel_Bbu_ during recovery from starvation. (XLSX)

S8 Table Genes downregulated by Rel_Bbu_ in stationary phase. (XLSX)

S9 Table Genes downregulated by Rel_Bbu_ during starvation. (XLSX)

S10 Table Genes downregulated by Rel_Bbu_ during recovery from starvation. (XLSX)

S11 Table Oligonucleotides used in this study. (DOCX)

S12 Table Genome coverage of data sets from RNA-seq. (DOCX)
