# Correction: The *Borrelia burgdorferi* RelA/SpoT Homolog and Stringent Response Regulate Survival in the Tick Vector and Global Gene Expression during Starvation

**DOI:** 10.1371/journal.ppat.1005242

**Published:** 2015-10-16

**Authors:** Dan Drecktrah, Meghan Lybecker, Niko Popitsch, Philipp Rescheneder, Laura S. Hall, D. Scott Samuels

The following information is missing from the Funding section: RNA-DK Biology grant W1207-B09. The correct funding information is:

This research was supported by the National Institute of Allergy and Infectious Diseases of the National Institutes of Health grant R01AI051486 (DSS), the National Center for Research Resources grant 5P20RR016455-11, RNA-DK Biology grant W1207-B09 and the National Institute of General Medical Sciences grant 8 P20 GM103474-11. The funders had no role in the study design, data collection and analysis, decision to publish, or preparation of the manuscript.
