# Supplementary figures and images for: The Borrelia burgdorferi RelA/SpoT Homolog and Stringent Response Regulate Survival in the Tick Vector and Global Gene Expression during Starvation
Source: PLoS Pathog. 2015 Sep 15;11(9):e1005160. doi: 10.1371/journal.ppat.1005160 (PMC4570706; doi:10.1371/journal.ppat.1005160)

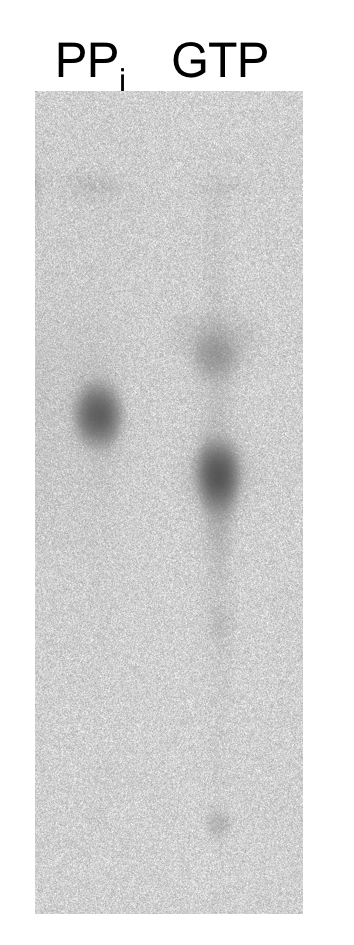

Supplement: S1 Fig — 32PPi and α-32P GTP were separated by TLC, plates were dried, exposed to a phosphor screen and visualized using a phosphorimager. (TIFF) [file ppat.1005160.s001.tiff]

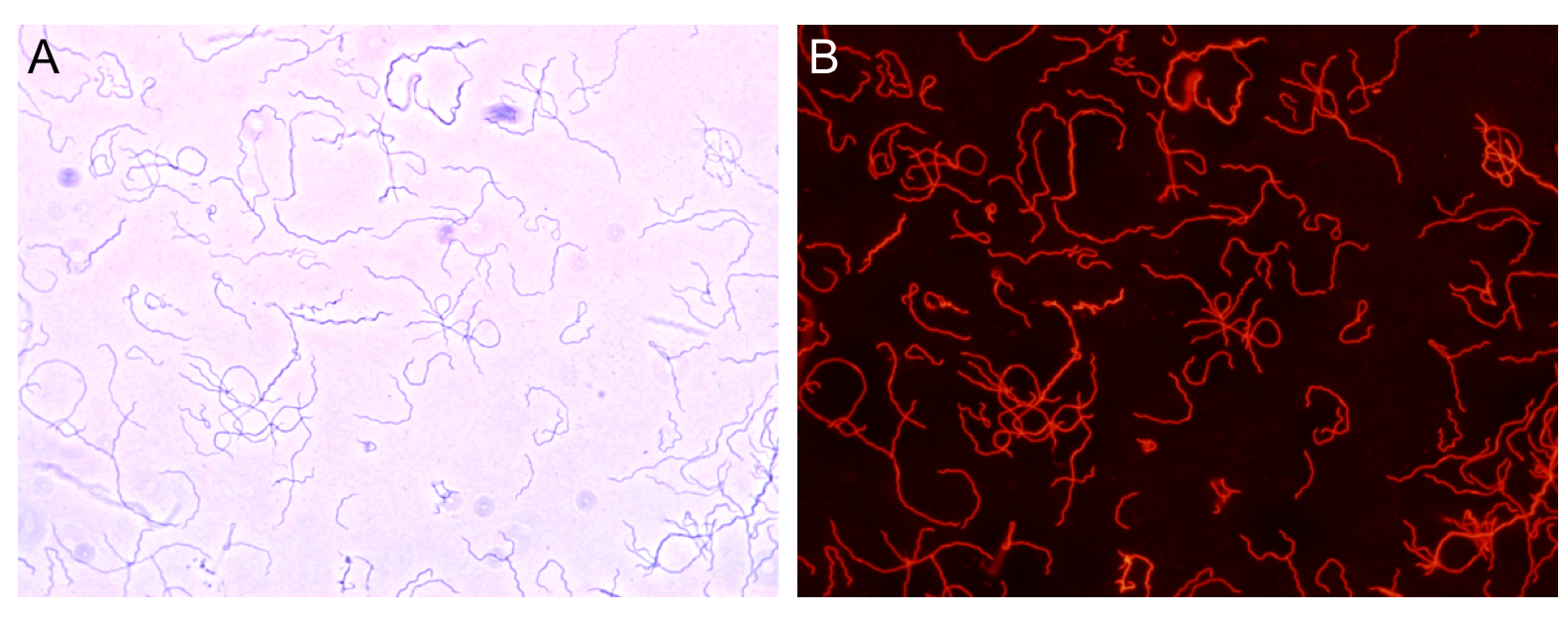

Supplement: S2 Fig — Wild-type B. burgdorferi grown in BSK + RS were incubated with WGA-Alexa Fluor 594 and visualized by DIC (A) and fluorescence microscopy (B). (TIFF) [file ppat.1005160.s002.tiff]

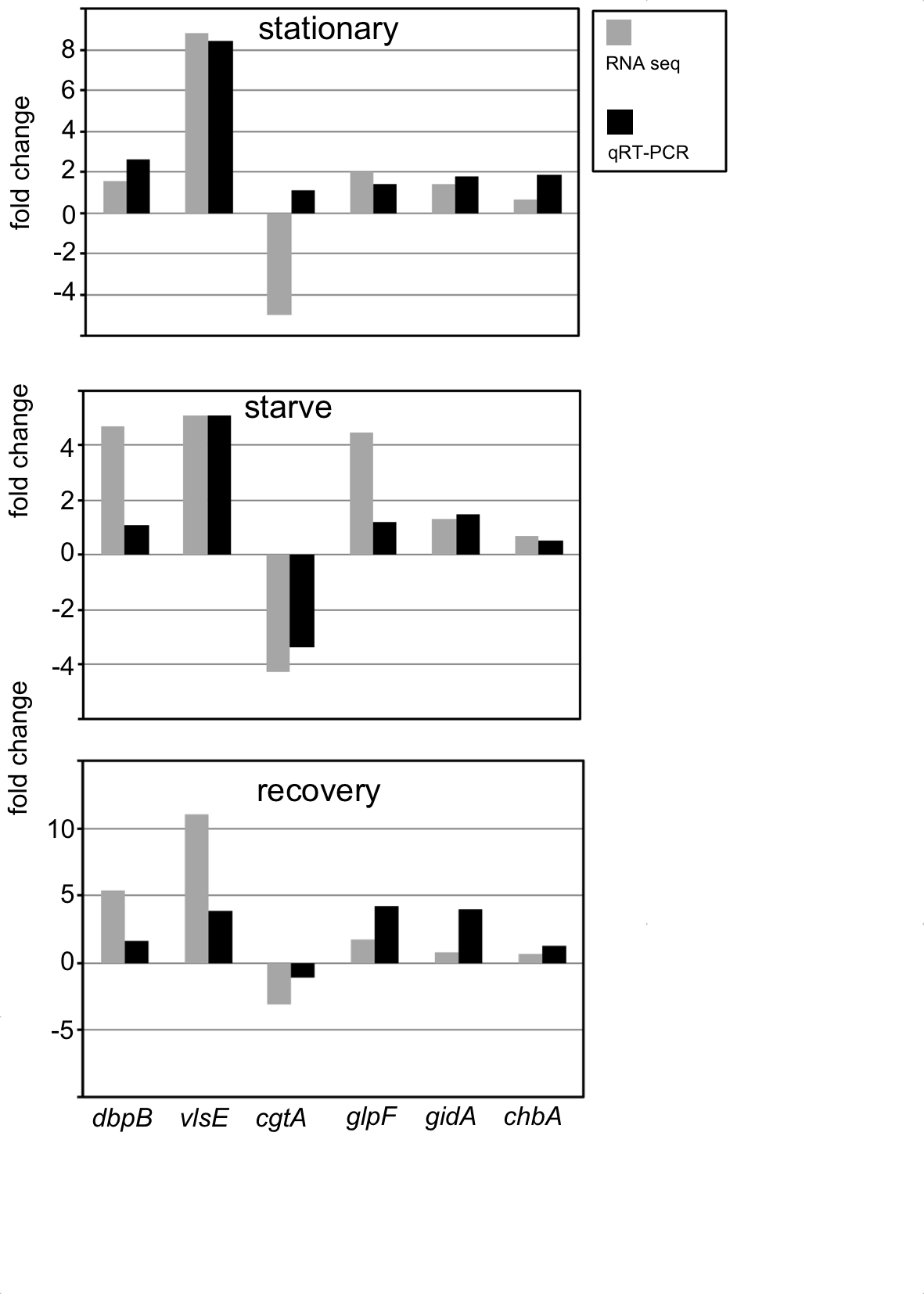

Supplement: S3 Fig — Fold changes represent transcript levels (wild type/rel Bbu mutant). Values for RNA seq are from S5–S10 Tables. TaqMan qRT-PCR was used for independent measurements of transcripts as described in the Materials and Methods for cultures grown to stationary phase, during starvation and recovery from starvation. qRT-PCR transcript levels were normalized to flaB levels. Values are the average of at least two independent experiments. The primers and probes used to measure transcript levels of dbpB, vlsE, cgtA, glpF, chbA and gidA are listed in S11 Table. (TIFF) [file ppat.1005160.s003.tiff]

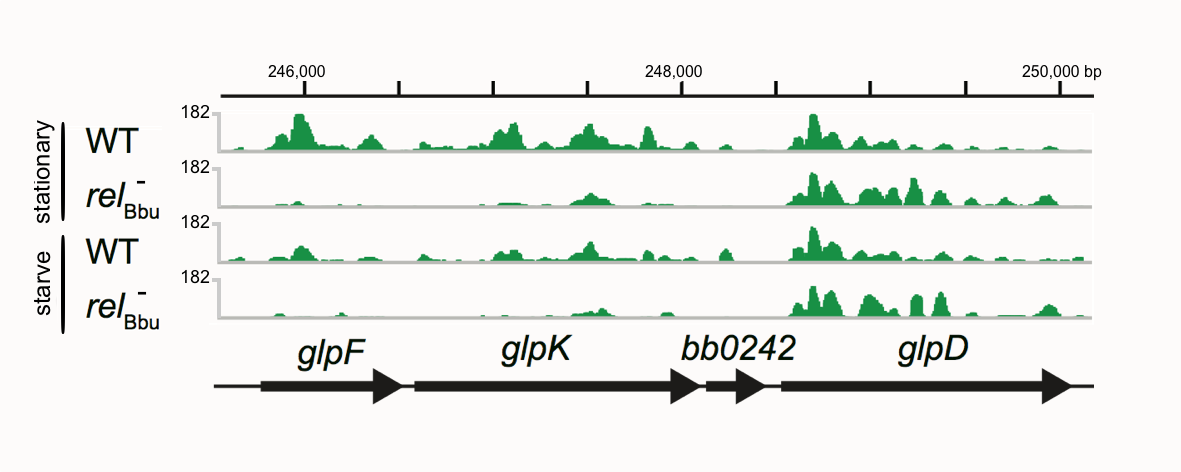

Supplement: S4 Fig — The RNA-seq results displayed in a coverage map of libraries in the wild-type and rel Bbu - mutant strains at stationary phase and starvation for the four genes of the glp operon. The height at each position indicates the number of reads that mapped to that base, with the highest read being 172. The genome context is depicted below the coverage maps. (TIFF) [file ppat.1005160.s004.tiff]

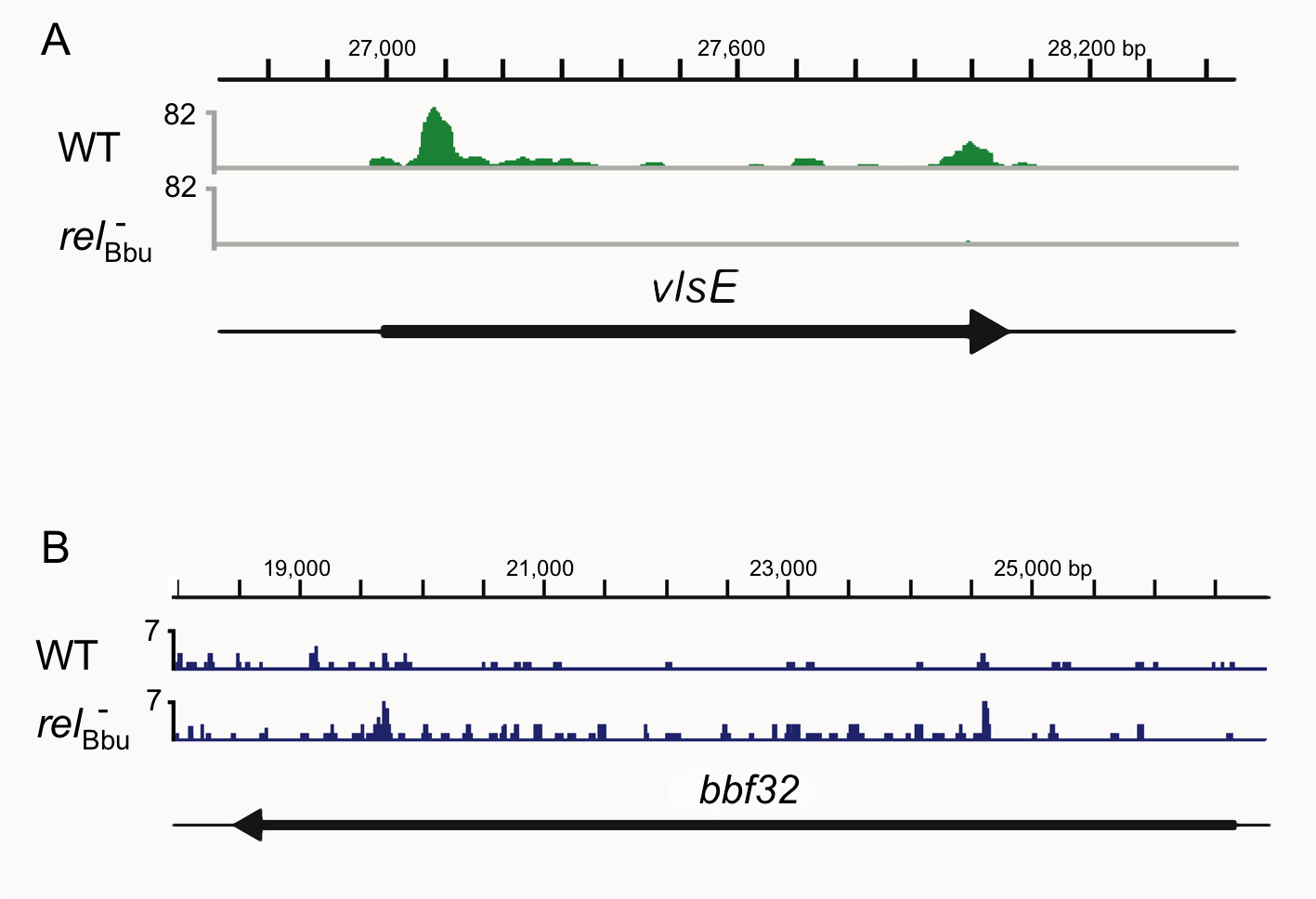

Supplement: S5 Fig — The RNA-seq results for (A) vlsE and (B) vls “silent” cassettes (bbf32) displayed in a coverage map of libraries in the wild-type and rel Bbu - mutant strains at stationary phase. The height at each position indicates the number of reads that mapped to that base, with the highest read being 82 in (A) and 7 in (B). The genome context is depicted below the coverage maps. (TIFF) [file ppat.1005160.s005.tiff]
