# Supplementary material for: The Borrelia burgdorferi RelA/SpoT Homolog and Stringent Response Regulate Survival in the Tick Vector and Global Gene Expression during Starvation
Source: PLoS Pathog. 2015 Sep 15;11(9):e1005160. doi: 10.1371/journal.ppat.1005160 (PMC4570706; doi:10.1371/journal.ppat.1005160)
Supplement: S11 Table — (DOCX) [file ppat.1005160.s016.docx]

**Table S11.** Oligonucleotides used in this study.

| Name | Sequence (5′ to 3′) |
| --- | --- |
| rsh U844F | AGAATTAAAAAGCAATAGCA |
| rsh 1F+NdeI | CATATGATACAAGCATACGAAAT |
| rsh 142R+Aat+Age | ACCGGTGAGGACGTCTGCTATTTCTAGAGCTTTG |
| rsh 1939F+AatII | GACGTCAAAGAAAACCCAAAT ATCTT |
| rsh D3102R+AgeI | ACCGGTAGCCTGCAAATATTTTATT |
| rsh U365F+AatII | GACGTCGCTCAAACTTGAAAAATTTG |
| rsh 2004R+AatII | GACGTCTTAATTGTCATAATTTTTAATGT |
| rsh 981F | AAGAATACCTGAGGATAACC |
| rsh 1984R | TGTCTTCTTCTATTTGCTT |
| flaB 423F | TTCTCAAAATGTAAGAACAGCTGAAGA |
| flaB 542R | TGGTTTGTCCAACATGAACTC |
| flaB probe | 6-FAM-TCACTTTCAGGGTCTCAAGCGTCTTGGAC-TAMRA |
| rsh 498F | TTTGCCCAAAAACAGACAAG |
| rsh 569R | AGGCGTTCTGCTATTGGAAC |
| rsh probe | 6-FAM-CAGGATTGCAAAAGATTGCCTTTCAAC-TAMRA |
| dbpB 149F | AAGCCACGGGAAAAGGTGT |
| dbpB 253R | TGGCTTGTACTTTTGCTTCTCT |
| dbpB probe | 6-FAM-ACCGGTTCCAAGGTAACAAGTGGTGGAC-TAMRA |
| vlsE 973F | ACTGGGCTAATAGGAGACGC |
| vlsE 1063R | TCAAGGCAGGAGGTGTTTCT |
| vlsE probe | 6-FAM-CCGGGCTAAGGAAAGTCGGTGATTCAGT-TAMRA |
| cgtA 553F | TCTAGGGTCGCAAATTATCCTTTT |
| cgtA 686R | GTCCCGAGCCCTACTCCAA |
| cgtA probe | 6-FAM-TTCCTCATCTTGGTATGCTCAGGCGTTC-TAMRA |
| glpF 166F | GGATTGGGTGTAACGTTTGGTATT |
| glpF 256R | CAACACTTGCTAATCCTATGCTAACAG |
| glpF probe | 6-FAM-CAGCAAGAATGAGCGGAGCACACCTAAAC-TAMRA |
| gidA 514F | AGGCTTGCTGAATTTTCTGCTTAT |
| gidA 652R | CAAATTGAACCTCCGTCTTTGAA |
| gidA probe | 6-FAM-AAAACGGGCACTCCAGCAAGAATTCATAAA-TAMRA |
| chbA 117F | CAAAGAATATGACAAGGCAGAGCTT |
| chbA 214R | TGGCTGATTGATGTATTATTTCTCTGT |
| chbA probe | 6-FAM-CCATTGCAAAGGCCCATGAAGCA-TAMRA |
