# Supplementary material for: The Borrelia burgdorferi RelA/SpoT Homolog and Stringent Response Regulate Survival in the Tick Vector and Global Gene Expression during Starvation
Source: PLoS Pathog. 2015 Sep 15;11(9):e1005160. doi: 10.1371/journal.ppat.1005160 (PMC4570706; doi:10.1371/journal.ppat.1005160)
Supplement: S12 Table — (DOCX) [file ppat.1005160.s017.docx]

Table S12. Genome coverage.

| **Sample** | **Sequenced** | **Mapped (%)** | **Coverage** | **Schutzer (%)** | **Coverage** |
| --- | --- | --- | --- | --- | --- |
| WT #1 37 A | 47,554,684 | 34,440,727 (72.42) | 1,132 | 2,544,677 (7.39) | 96 |
| WT #1 37 B | 47,304,762 | 33,936,290 (71.74) | 1,115 | 2,502,580 (7.37) | 94 |
| WT #1 37 C | 42,969,592 | 32,859,070 (76.47) | 1,080 | 4,891,586 (14.89) | 185 |
| WT #2 37 A | 42,043,380 | 29,037,749 (69.07) | 954 | 2,658,763 (9.16) | 100 |
| WT #2 37 B | 39,309,059 | 28,133,741 (71.57) | 925 | 2,203,387 (7.83) | 83 |
| WT #2 37 C | 36,689,473 | 26,024,680 (70.93) | 855 | 3,696,339 (14.20) | 139 |
| rshKO #1 37 A | 31,318,947 | 26,074,781 (83.26 | 857 | 2,586,599 (9.92) | 98 |
| rshKO #1 37 B | 36,327,517 | 31,379,208 (86.38) | 1,031 | 1,959,977 (6.25) | 74 |
| rshKO #1 37 C | 35,915,909 | 29,344,543 (81.70) | 965 | 4,313,763 (14.70) | 163 |
| rshKO #2 37 A | 37,632,548 | 31,078,606 (82.58) | 1,022 | 4,408,560 (14.19) | 166 |
| rshKO #2 37 B | 43,611,699 | 37,168,422 (85.23) | 1,222 | 3,045,490 (8.19) | 115 |
| rshKO #2 37 C | 48,261,725 | 41,645,200 (86.29) | 1,369 | 3,061,755 (7.35) | 115 |
| rshKO Comp#1 A | 47,615,152 | 38,429,589 (80.71) | 1,263 | 6,176,817 (16.07) | 233 |
| rshKO Comp #1 B | 51,767,758 | 40,299,680 (77.85) | 1,325 | 4,975,778 (12.35) | 188 |
| rshKO Comp #1 C | 46,848,270 | 37,899,777 (80.90) | 1,246 | 3,522,617 (9.29) | 133 |
| rshKO Comp #2 A | 44,746,214 | 35,034,365 (78.30) | 1,152 | 5,294,949 (15.11) | 200 |
| rshKO Comp #2 B | 41,848,731 | 32,039,581 (76.56) | 1,053 | 3,712,592 (11.59) | 140 |
| rshKO Comp #2 C | 42,666,469 | 33,313,638 (78.08) | 1,095 | 2,998,008 (9.00) | 113 |
